# Increased expression of miR-194-5p through the circPVRL3/miR-194-5p/SOCS2 axis promotes proliferation and metastasis in pancreatic ductal adenocarcinoma by activating the PI3K/AKT signaling pathway

**DOI:** 10.1186/s12935-022-02835-0

**Published:** 2022-12-20

**Authors:** Bojing Chi, Yao Zheng, Fuming Xie, Wen Fu, Xianxing Wang, Jianyou Gu, Jiali Yang, Jingyang Yin, Lei Cai, Peng Tang, Jianbo Li, Shixiang Guo, Huaizhi Wang

**Affiliations:** 1 Savaid Medical School, University of Chinese Academy of Sciences, Beijing, 100049 People’s Republic of China; 2 Institute of Hepatopancreatobiliary Surgery, Chongqing General Hospital, Chongqing, 401147 People’s Republic of China; 3 Chongqing Key Laboratory of Intelligent Medicine Engineering for Hepatopancreatobiliary Diseases, Chongqing, 401147 People’s Republic of China; 4 Chongqing Medical University, Chongqing, 400016 People’s Republic of China; 5 College of Life Sciences, University of Chinese Academy of Sciences, Beijing, 100049 People’s Republic of China

**Keywords:** Circular RNA, miR-194-5p, Cell cycle, Pancreatic ductal adenocarcinoma

## Abstract

**Background:**

MicroRNAs (miRNAs), as an indispensable type of non-coding RNA (ncRNA), participate in diverse biological processes. However, the specific regulatory mechanism of certain miRNAs in pancreatic ductal adenocarcinoma (PDAC) remains unclear.

**Methods:**

The expression of miR-194-5p in PDAC tissue microarray and cell lines were detected by RNA-scope and real-time quantitative PCR (RT-qPCR). The function of proliferation and migration carried by miR-194-5p in vitro and vivo was observed by several functional experiments. Informatics methods and RNA sequencing data were applied to explore the target of miR-194-5p and the upstream circular RNA (circRNA) of miR-194-5p. RNA-binding protein immunoprecipitation (RIP) assay and dual-luciferase reporter assay confirmed the relationships between miR-194-5p and SOCS2 or miR-194-5p and circPVRL3. The proliferation and migration abilities of SOCS2 and circPVRL3 were accessed by rescue experiments.

**Results:**

In this study, we aimed to clarify the molecular mechanisms of miR-194-5p, which has critical roles during PDAC progression. We found that the expression of miR-194-5p was significantly upregulated in PDAC tissue compared to tumor-adjacent tissue and was highly related to age and nerve invasion according to RNAscope and RT‒qPCR. Overexpression of miR-194-5p accelerated the cell cycle and enhanced the proliferation and migration processes according to several functional experiments in vitro and in vivo. Specifically, circPVRL3, miR-194-5p, and SOCS2 were confirmed to work as competing endogenous RNAs (ceRNAs) according to informatics methods, RIP, and dual-luciferase reporter assays. Additionally, the rescue experiments confirmed the relationship among miR-194-5p, circPVRL3, and SOCS2 mRNA. Finally, the circPVRL3/miR-194-5p/SOCS2 axis activates the PI3K/AKT signaling pathway to regulate the proliferation and metastasis of PDAC.

**Conclusion:**

Our findings indicated that an increase of miR-194-5p caused by circPVRL3 downregulation stimulates the PI3K/AKT signaling pathway to promote PDAC progression via the circPVRL3/miR-194-5p/SOCS2 axis, which suggests that the circPVRL3/miR-194-5p/SOCS2 axis may be a potential therapeutic target for PDAC patients.

**Supplementary Information:**

The online version contains supplementary material available at 10.1186/s12935-022-02835-0.

## Introduction

PDAC is a fatal type of cancer worldwide; it has a high mortality rate and a 5-year survival rate of less than 5% [1]. Among malignant tumors, PDAC is the fourth leading cause of death among malignant tumors in the United States [2]. In recent years, the incidence and mortality rates have shown an increasing trend in China [3]. Some studies predict that the global fatality rate will be the second highest in the world by 2030 [4, 5]. Although the understanding of PDAC is gradually deepening, the pathogenesis remains unclear. The serum level of CA19-9 is the most commonly used diagnostic marker for PDAC, but the current screening methods cannot achieve effective early diagnosis [6]. In addition, patients are still mostly treated with surgeries and diagnosis relies heavily on imaging technology, which is the main constraint on their prognosis. Therefore, exploring the novel mechanisms of PDAC is an effective way to seek new drug targets and therapeutic strategies.

MiRNAs are a crucial cluster of ncRNA which are 21–25 nucleotides long [7]. They have the potential to be therapeutical for disease genes by complementing the 3’UTR of mRNAs and forming an RNA-induced silencing complex (RISC) to block the activity of gene transcription [8–10]. Furthermore, miRNAs can regulate various biological processes such as proliferation, metastasis, apoptosis, and DNA damage by regulating the expression of target genes [11–14]. Recently, some studies have shown promise in suppressing tumor progression [15–17]. For instance, it is well known that mutations in KRAS contribute to PDAC tumorigenesis, but there are no effective therapeutic ways of targeting KRAS until now. Hence, the emergence of miRNAs compensates for the lack of drugs by targeting mutant KRAS such as miR-96, miR-126, and miR-143 which showed an inhibitory effect by targeting KRAS to regulate RAS family proteins [18]. However, the mechanisms of miRNAs during PDAC progression are still elusive.

The suppressor of cytokine signaling 2 (SOCS2) is a member of the suppressor of cytokine signaling (SOCS) family. SOCS2 plays a tumor suppressor role in multiple tumors. Previous studies have shown that inhibiting SOCS2 can promote the proliferation and metastasis of colon cancer [19, 20]. SOCS2 is also a target of several miRNAs such as miR-196b, miR-301a-3p, and miR-3648, which regulate the proliferation and metastasis of malignant tumors [21–23]. However, the molecular mechanisms of SOCS2 in PDAC tumorigenesis are still obscure.

CircRNAs are another type of functional ncRNA generated by back-splicing and covalent linking with the 3’ splice site and the 5’ splice site [24, 25]. In recent years, many studies have shown that circRNAs are aberrantly expressed in various cancers [26–29]. CircRNAs can combine with miRNAs through miRNA recognition elements (MREs) [30]. Therefore, circRNA sponges may be an effective mechanism to play an important role in tumors relying on ceRNAs surrounding circRNAs, miRNAs, and targets [24, 25, 31]. Moreover, circRNAs function as miRNA sponges in many facets of PDAC such as proliferation, metastasis, angiogenesis, and apoptosis [32].

In the present study, our results indicated that miR-194-5p was significantly upregulated in PDAC tissue compared to tumor-adjacent tissue, and clarified the underlying mechanism by which circPVRL3 competitively absorbs miR-194-5p as miRNA sponge to regulate the expression of the target gene SOCS2 and activate the PI3K/AKT signaling pathway to promote the progression of PDAC.

## Materials and methods

### Cell culture

All PDAC cell lines (PANC-1, BxPC-3, AsPC-1, CFPAC-1 and SW1990), and HPDE-C7 were purchased from American Type Culture Collection (ATCC). BxPC-3 cells were cultured in RPMI-1640 medium (Gibco, USA), HPDE-C7 cells were cultured in minimum essential medium (MEM) (HyClone, USA) and SW1990 cells were cultured in Leibovitz's L-15 medium with 10% fetal bovine serum (FBS) (HyClone, USA) and 1% penicillin–streptomycin solution (Solarbio, China). The remaining cell lines were cultured in the Dulbecco’s modified Eagle’s medium (DMEM) (Gibco, USA) medium with 10% FBS and 1% penicillin–streptomycin solution. All cell lines were maintained at 37 °C in a humidified atmosphere containing 5% CO_2_.

### Transfection and cell treatment

Plasmids and small interfering RNA (siRNA) transfections were performed using Lipofectamine 3000 (Invitrogen, USA) according to the manufacturer’s instructions. Hsa-miR-194-5p mimic or mimic NC miRNA (RiboBio, China) was transfected into cells at a final concentration of 50 nM; hsa-miR-194-5p inhibitor or inhibitor NC miRNA (RiboBio, China) was transfected into cells at a final concentration of 200 nM. The plasmids, pcDNA3.1-SOCS2 (GenePharma, China) and pLC5-ciR-circPVRL3 (GENESEED, China) were transfected (2.5 µg) into cells. si-SOCS2 and si-circPVRL3 (RiboBio, China) were transfected into cells at a final concentration of 100 nM. The sequences of the siRNAs were listed in Additional file 1: Table S3. For lentivirus transduction, 10^6^ PDAC cells were incubated in a 6-well plate with 1 ml of medium containing 10 µl (10^8^ U) of lentivirus particles and 5 µg/ml polybrene for 24 h.

### CCK-8 assay

Cells were seeded at 5 × 10^3^ cells per well in the 96-well plates. All groups were examined at 1 to 5 days by using a CCK-8 (Bioground, China) assay, and the absorbance was measured at 450 nm with a microplate reader. All assays were repeated 3 times.

### EdU proliferation assay

To visualize cell proliferation, cells transfected with miR-194-5p mimic or inhibitor were cultured on 24-well plates, and cells were incubated with the thymidine analog EdU at a final concentration of 100 µM for 2 h. Then, the cells were fixed with 4% paraformaldehyde and an EdU proliferation assay was performed using the EdU Cell Proliferation Kit (BeyoClick™ EdU-594 #C0078L, Beyotime, China) following the manufacturer’s protocol. Cells were observed by immunofluorescence microscopy (Olympus, Tokyo, Japan).

### Flow cytometry for cell cycle analysis

Transfected cells in 6-well plates were digested with trypsin and fixed with 70% ethyl alcohol at 4 °C overnight. The next day, the cells were washed once with PBS and resuspended in 1 ml PBS, and then stained using the propidium iodide method following the manufacturer’s protocol (Beyotime, China). Cell cycle analysis was performed by using a FACSCalibur FACS scanner (Becton Dickinson).

### Colony formation assay

The transfected cells were seeded in the 6-well plates at 10^3^/well. After 10–14 days of cell culture, the cells were fixed with 4% paraformaldehyde and stained with crystal violet (Beyotime, China). The number of colonies was analyzed by the ImageJ software (National Institutes of Health, Bethesda, MD).

### Wound-healing and Transwell migration assays

For the wound healing assay, cells were seeded into 6-well plates after transfection, and the cell monolayer was scratched with a 10 µl sterile micropipette tip to create artificial wounds. Representative images were captured at 0 and 24 h. For Transwell migration assays, were performed using cell culture inserts with 8 μm pore size transparent PET membranes (Corning, MA, USA). A total of 5 × 10^4^ cells were resuspended in serum-free medium and placed into the upper chamber, and medium supplemented with 10% FBS was added to the lower chamber. After 10 h or 12 h, the migrated cells were fixed with 4% paraformaldehyde and stained with crystal violet (Beyotime, China), and the migrated cells were counted by ImageJ software.

### Real-time quantitative PCR assays and RNase R treatment

Total RNA extracted with a TRIzol Reagent (Bioground, China) was reverse‐transcribed according to the procedures of a PrimeScript™ RT reagent Kit with gDNA Eraser Kit (Takara, Kusatsu, Japan). For miRNA reverse‐transcribed reactions, the procedures followed the TaqMan™ MicroRNA Reverse Transcription Kit (#4366596, Thermo Fisher Scientific, USA). The reverse‐transcribed reactions were performed on a Thermal Cycler Dice instrument (Bio-Rad Laboratories Inc., Hercules, CA, USA). mRNA expression was quantified with an SYBR Premix Ex Taq II (Takara, Kusatsu, Japan) and miRNA expression was quantified with a TaqMan Universal Mastermix II, no UNG kit (#4440040, Thermo Fisher Scientific, USA). The real‐time quantitative PCR(RT‒qPCR) system reaction conditions were as follows: 95 °C for 30 s, 95 °C for 5 s, and 60 °C for 34 s with 40 cycles for mRNA; 95 °C for 10 min, 95 °C for 15 s, and 60 °C for 1 min for miRNA. Primers for hsa-miR-194-5p and U6 reverse‐transcribed and amplified were purchased from Thermo Fisher (Thermo Fisher Scientific, USA). The U6 or GAPDH was used for normalization, and all of the reactions were performed in triplicate. The relative levels of gene expression were calculated using the 2^−ΔΔCt^ method. Divergent primers and convergent primers were used to validate circRNA. Primer sequences are listed in Additional file 1: Table S2. For RNase R treatment, 5 U RNase R was added to the digest 1 μg of RNA for 30 min at 37 °C. The relative RNA levels were examined by agarose gel electrophoresis.

### Western blotting

Total protein was extracted from transfected cells that were lysed with RIPA buffer (Solarbio, China) containing the protease inhibitor PMSF. Proteins were separated by SDS‒PAGE and blotted onto PVDF membranes. Primary antibodies against P21, E-cadherin, N-cadherin, CDK2, CDK4, Cyclin D1, Cyclin E1, p-AKT, AKT, p-PI3K, PI3K, GAPDH (1:1000; Cell Signaling Technology) and SOCS2(1:1000; ABclonal) were incubated overnight at 4 °C. Following incubation with secondary HRP-conjugated antibodies (1:5000; Cell Signaling Technology), the membranes were incubated for 1 h at room temperature. The chemiluminescence signal was detected using an ECL chemiluminescence system (Bioground, China).

### Luciferase reporter assay

The wild-type (WT) sequence and mutant-type (Mut) of the circPVRL3 as well as the WT and Mut 3ʹUTR sequences of the SOCS2 that contained the predicted binding site of the hsa-miR-194-5p were subcloned them into the GP-miRGLO reporter vectors (constructed by GenePharma, China). A Dual-Luciferase Reporter System (Promega, USA) was applied to examine relative luciferase activities.

### RNAscope assay

The expression of hsa-miR-194-5p in the tissue microarray was tested by using the RNAScope 2.5 HD-RED assay (324500, Advanced Cell Diagnostics, Newark, CA) following the manufacturer’s protocol. The probes used included the positive control probe U6 (727871-S1) (positive control, 313901) and the negative control probe scramble (727881-S1) (negative control, 310043) (Advanced Cell Diagnostics, Newark, CA).

### Fluorescence in situ hybridization (FISH)

Cells were seeded in the confocal dishes at 1 × 10^4^ cells per dish. Cells were fixed with 4% paraformaldehyde. For the FISH assay, Cy3 labeled cirPVRL3 (hsa_circ_0004639) and FAM labeled hsa-miR-194-5p were synthesized by GenePharma. The probe signals were observed by the RNA FISH Kit (GenePharma, China) following the manufacturer’s instructions. Images were captured by a Leica confocal microscope. The probe sequence of circPVRL3 was: 5′-GCTAAGGCACCTGCTCCACACAGAT-3′, and that of miR-194-5p was:5′-TCCACATGGAGTTGCTGTTACA-3′.

### mRNA-sequencing (mRNA-seq) and bioinformatics analysis

Total RNA was extracted from two groups of PANC-1 cells transfected with miR-194-5p mimic or mimic NC miRNA, and the experiment was repeated three times to obtain three biological replicates The mRNA-seq was implemented by the DNBSEQ platform (BGI company, Shenzhen, China), and subsequent data were analyzed by Dr.Tom system (BGI, Shenzhen, China). We used the miRNA target gene prediction websites TargetScan (http://www.targetscan.org/vert_72/) [33], miRDB (http://mirdb.org/) [34], StarBase (http://starbase.sysu.edu.cn) [35], Tarbase (http://carolina.imis.athena-innovation.gr/diana_tools/web/) [36] and miRPathDB (https://mpd.bioinf.uni-sb.de/) [37]. We also used the public datasets GSE43795 and GSE79634 to assist in identifying the upstream and downstream targets of miR-194-5p.

### RIP assay

RIP assays were performed by a Magna RIP™ Kit (Millipore, Burlington MA, USA) according to the manufacturer's protocol. Cells were lysed in RIP lysis buffer, and RIP lysate was incubated with the magnetic beads conjugated with AGO2 (Abcam ab186733) or IgG antibody and rotated overnight at 4 °C. The immunoprecipitation products were pulled down by the bead-antibody complex and washed with wash buffer. Immunoprecipitated RNAs were analyzed by RT‒qPCR and normalized to the input control.

### Animal xenograft model

Animal experiments were approved by the Ethics Committee of Chongqing General Hospital. Four-week-old nude mice were used in the study. Approximately 5 × 10^6^ PANC-1 or CFPAC-1 cells were injected into the armpits of nude mice subcutaneously (5 mice per group). When the tumor grew up to 5 mm × 5 mm (approximately 2 weeks), it was injected with 1 nmol miR-194-5p agomir or 5 nmol antagomir with a multipoint injection of the tumor. For the rescue animal models, nude mice were subcutaneously injected with 5 × 10^6^ with Lv-circPVRL3 or Lv-NC (Cyagen, China) with CFPAC-1 cells into the armpits of nude mice subcutaneously and treated with/without miR-194-5p agomir/antagomir following the methods above. The tumor size was measured every 7 days, and the mice were sacrificed at 1 month after injection. The tumors were fixed in 4% paraformaldehyde and embedded in paraffin.

### Immunohistochemistry

Paraffin sections of tumor tissues were cut at a thickness of 5 μm. Sections were incubated with the primary antibody anti-Ki-67 (CST, #9949, 1:200); P21 (CST, #2947, 1:50); E-cadherin (Proteintech, #20874-1-AP, 1:2000); N-cadherin (Proteintech, #22018-1-AP, 1:1000) at 4 °C overnight. Sections were incubated with HRP‐polymer‐conjugated secondary antibodies after washing with phosphate‐buffered saline, and then they were immunostained using a DAB plus kit (ZSGB-Bio, Beijing, China).

### Statistical analysis

All data are presented as the mean ± standard deviation (SD). Evaluation of clinical characteristics was performed by the chi-square test and differences between the two groups were analyzed using Student’s t-test. The paired samples were subjected to a paired-samples t-test. All statistical analyses were performed with the SPSS 20.0 software (IBM Corp., Armonk, NY, USA), and two-tailed *P* values of < 0.05 were defined as statistically significant. The degree of significance is expressed as: ^*^p < 0.05, ^**^p < 0.01, or ^***^p < 0.001.

## Results

### The expression of miR-194-5p is significantly increased in PDAC

**Fig. 1:**
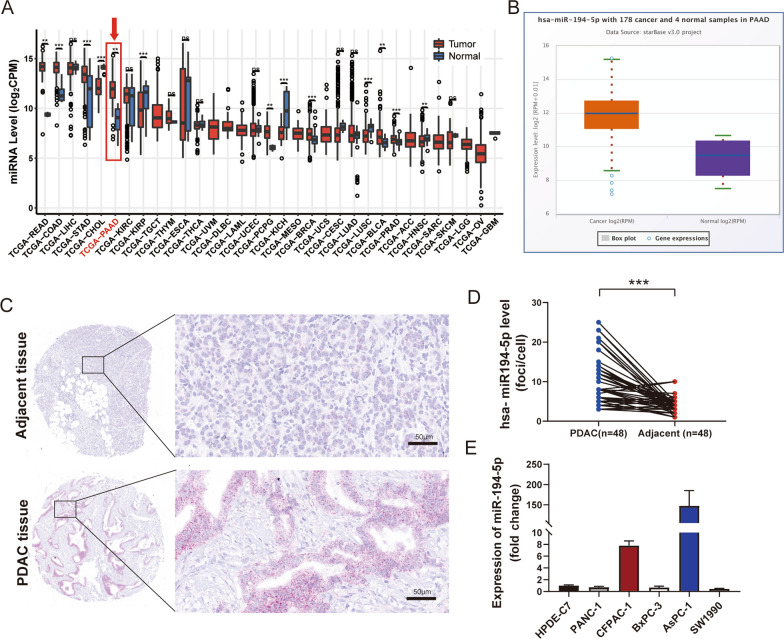
Expression of miR-194-5p in PDAC tissues and PDAC cell lines. **A** The bar graph shows the expression of miR-194-5p in tumor and normal tissues of various tumors according to TCGA data. **B** The expression of miR-194-5p in PDAC on the basis of the TCGA database. **C** Representative pictures showing the expression of miR-194-5p in PDAC tissues and tumor-adjacent tissues examined by RNAscope assay, and it is represented by red dots. Magnification 400×, scale bar = 50 μm. **D** The expression of miR-194-5p in 48 paired PDAC tissues and tumor-adjacent tissues was used paired Student's t-test for the statistical analyses. **E** The expression level of miR-194-5p in normal pancreas cells and several PDAC cell lines was investigated by real-time quantitative PCR. *p < 0.05; **p < 0.01; ***p < 0.001; ns, not significant

MiR-194-5p expression was significantly increased in PDAC tissue compared to the normal tissue, which was consistent with the data from The Cancer Genome Atlas dataset (TCGA) (Fig. 1A, B). Additionally, we performed RNA hybridization in situ to evaluate the difference between PDAC and tumor-adjacent tissue derived from tissue microarray containing 58 paired PDAC samples and 12 PDAC samples (Fig. 1C). We detected 48 paired PDAC samples and the results demonstrated that miR-194-5p upregulated in PDAC according to RNAscope (Fig. 1D). The basic information and clinicopathological features of patients in the tissue microarray were summarized in Additional file 1: Table S1. Next, a correlation analysis of miR-194-5p expression and clinicopathological features of PDAC was performed. We statistically analyzed data for 56 cases of PDAC patients who were divided into two groups according to the RNAscope score: the high expression group (n = 22) and the low expression group (n = 34). We found that overexpression of miR-194-5p was significantly correlated with age and nerve invasion in PDAC patients (Table 1).

**Table 1:** Correlations between miR-194-5p and clinicopathologic features in PDAC

Clinicopathological	miR-194-5p expression		*p* value
Characteristics	Low(n = 34)	High(n = 22)	
Age (years)			**0.028**
≥ 60	18 (32%)	18 (32%)	
< 60	16 (29%)	4 (7%)	
Gender			0.480
Female	14 (25%)	7 (12%)	
Male	20 (36%)	15 (27%)	
Clinical stage			0.329
I	20 (36%)	13 (23%)	
II	6 (11%)	7 (12%)	
III	5 (9%)	2 (4%)	
IV	3 (5%)	0 (0%)	
T classification			0.387
T1	2 (4%)	0 (0%)	
T2	26 (46%)	16 (28%)	
T3	6 (11%)	6 (11%)	
N classification			0.436
N0	23 (41%)	17 (30%)	
N1	11 (20%)	5 (9%)	
Metastasis			0.152
Yes	31 (55%)	22 (40%)	
No	3 (5%)	0 (0%)	
Pathology grade			0.224
I	3 (5%)	4 (7%)	
II	20 (36%)	15 (27%)	
III	11 (20%)	3 (5%)	
Nerve invasion			**0.021**
Yes	27 (48%)	11 (20%)	
No	7 (12%)	11 (20%)	

Bold values represent statistically significant *p*–value

According to the above findings, we verified the expression of miR-194-5p in five PDAC cell lines (PANC-1, AsPC-1, BxPC-3, CFPAC-1, SW1990) and a normal human pancreatic duct epithelial cells (HPDE6-C7) by RT‒qPCR (Fig. 1E). The results demonstrated that miR-194-5p was highly expressed in AsPC-1 and CFPAC-1 cells versus the HPDE6-C7 cells, and the expression in other cell lines were similar to the HPDE6-C7 cells.

### MiR-194-5p promotes the proliferation and migration of PDAC cells

**Fig. 2:**
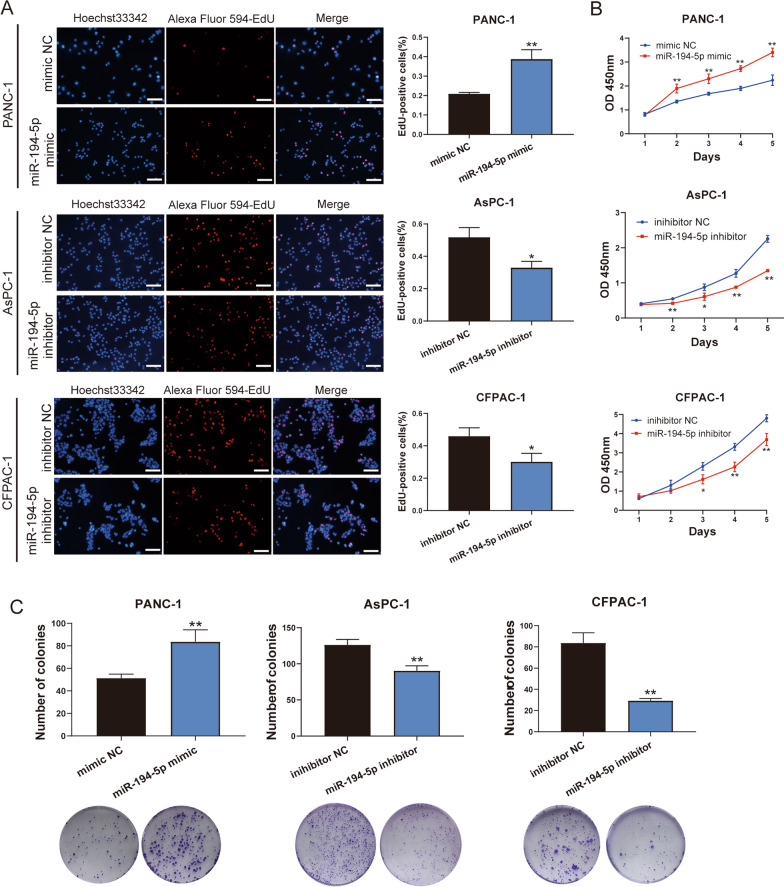
MiR-194-5p promotes the proliferation of PDAC cells. **A** EdU assay and CCK-8 assay **B** showed that overexpression of miR-194-5p accelerated DNA replication and promoted the proliferation of PDAC cells; knockdown of miR-194-5p inhibited these effects. Magnification 200×, scale bar = 100 μm. **C** The colony formation ability after treatment with miR-194-5p mimic or its inhibitor was evaluated by a colony formation assay

**Fig. 3:**
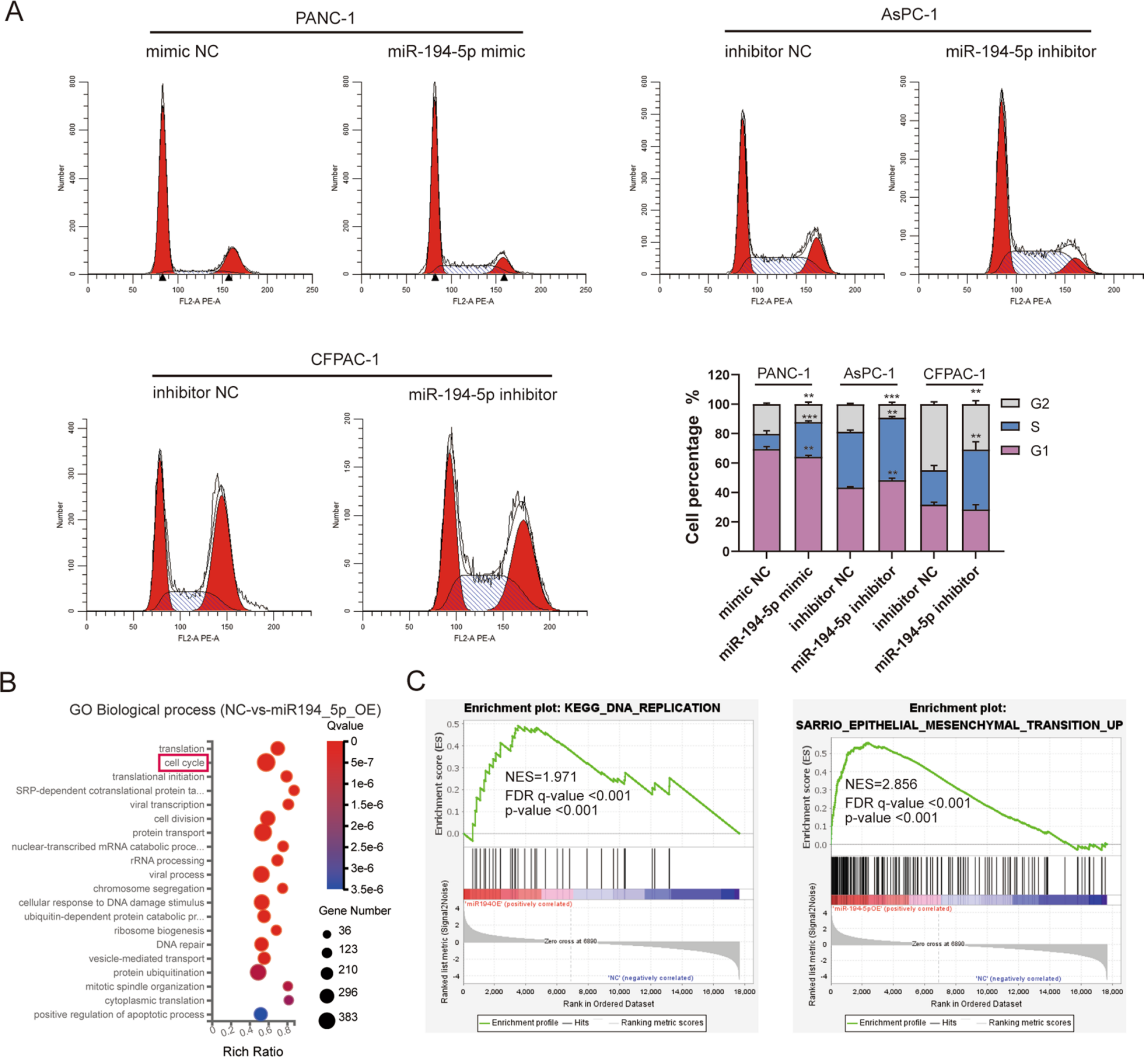
MiR-194-5p accelerates cell cycle and it is related to DNA replication and EMT. **A** A cell cycle assay was performed by flow cytometry to detect the characteristics of the specific phase of the cell cycle affected by transfection with miR-194-5p mimic or its inhibitor. **B**, **C** GO enrichment of biological processes and GSEA enrichment analyses were analyzed according to mRNA-seq data. *p < 0.05; **p < 0.01; ***p < 0.001

To explore the biological function of miR-194-5p in PDAC development and progression, miR-194-5p was overexpressed in PANC-1 cells and knocked down in CFPAC-1 and AsPC-1 cells. The transfection efficiency was evaluated by RT‒qPCR (Additional file 2: Fig. S1A). CCK-8, EdU, and colony formation assays showed that overexpression of miR-194-5p promoted cell proliferation and DNA replication, in contrast, miR-194-5p knockdown induced an inhibitory effect (Fig. 2A–C). Furthermore, we performed flow cytometry to observe the cell cycle. The results confirmed that overexpression of miR-194-5p increased the percentage of cells in the S phase, and miR-194-5p knockdown blocked both G0/G1 and S phase in AsPC-1 cells and S phase in CFPAC-1 cells (Fig. 3A). Gene ontology (GO) enrichment of biological processes and the Kyoto Encyclopedia of Genes and Genomes (KEGG) pathway enrichment also confirmed that genes related to overexpression of miR-194-5p were significantly enriched in cell cycle processes (Fig. 3B, Additional file 2: Fig. S1C), and gene set enrichment analysis (GSEA, http://software.broadinstitute.org/gsea/index.jsp) [38, 39] were revealed that genes related to the overexpression of miR-194-5p in the mRNA-seq analysis were closely correlated with DNA replication and epithelial-mesenchymal transition (EMT) according to the mRNA-seq data (Fig. 3C).

**Fig. 4:**
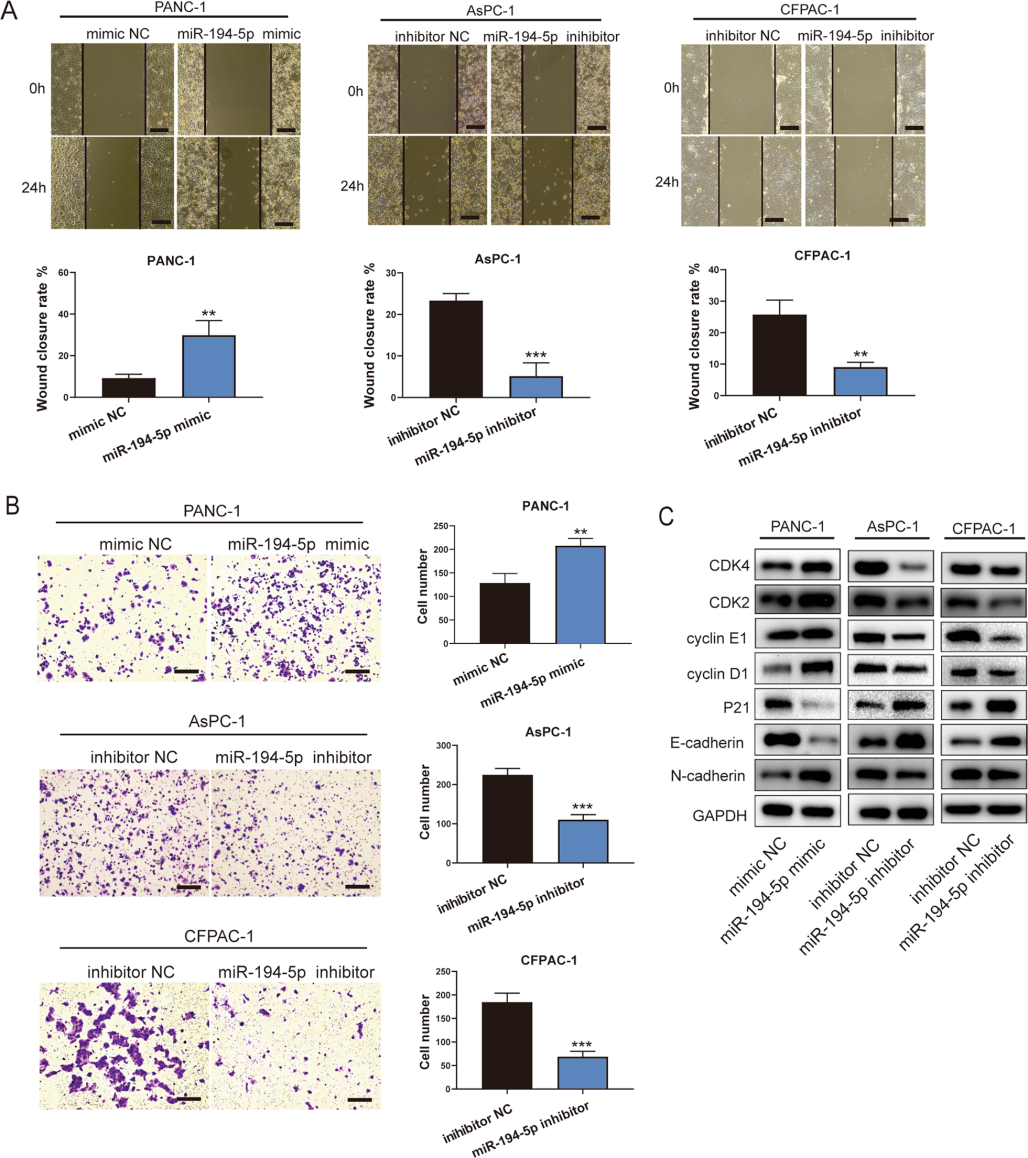
MiR-194-5p promotes the migration of PDAC cells in vitro. **A**, **B** the migration ability of PDAC cells transfected with miR-194-5p mimic or its inhibitor was assessed by wound healing assay and Transwell assay. Magnification 100×, scale bar = 200 μm. **C** The main biomarkers of cell cycle, proliferation and migration were detected by western blotting. *p < 0.05; **p < 0.01; ***p < 0.001

Subsequently, we performed wound healing assay and Transwell assays to examine the migration capability. These results indicated that overexpression of miR-194-5p enhanced cell migration whereas downregulation of miR-194-5p induced the opposite effect (Fig. 4A, B). Furthermore, we verified the markers of the cell cycle and EMT. The results showed that overexpressed miR-194-5p significantly increased the expression of cyclin D1, cyclin E1, CDK2, CDK4, and decreased the expression of P21 sharply. Additionally, overexpression of miR-194-5p increased the expression of N-cadherin and decreased the expression of E-cadherin. These effects were reversed in AsPC-1 and CFPAC-1 cells treated with the miR-194-5p inhibitor (Fig. 4C). In summary, these results indicated that miR-194-5p promoted the proliferation and migration of PDAC cells.

### Overexpression of miR-194-5p promotes PDAC tumor growth in vivo

**Fig. 5:**
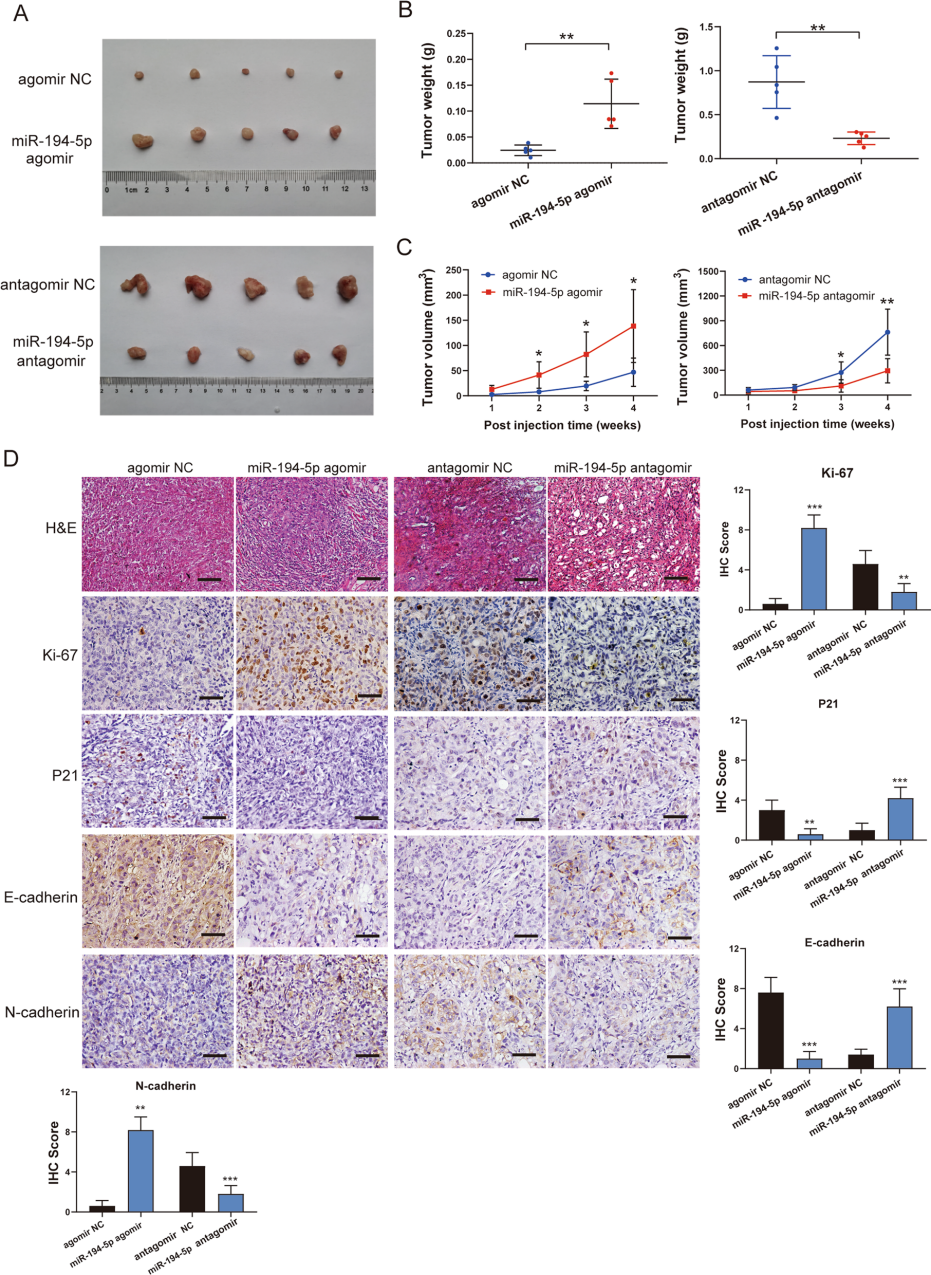
The overexpression or knockdown of miR-194-5p affects PDAC tumor progression in vivo. **A** Picture of the tumors obtained from the animal xenograft model., which was established through subcutaneous injection of PANC-1 and CFPAC-1 cells and subsequent treatment with miR-194-5p agomir, antagomir, agomir NC or antagomir NC (n = 5). **B** The tumor weights for each group were recorded at the endpoint. **C** The weekly changes in tumor volume were recorded and compared to the changes in the negative control groups. **D** HE staining of tumors derived from the subcutaneous xenograft model with magnification 200×, scale bar = 100 μm. The expression of Ki-67, P21, E-cadherin and N-cadherin was determined by IHC staining at a magnification of 400×, scale bar = 50 μm (n = 5). IHC scoring was used for the evaluation of IHC staining. *p < 0.05; **p < 0.01; ***p < 0.001

To investigate the role of miR-194-5p in tumor growth in vivo, PANC-1 cells treated with miR-194-5p agomir or agomir NC and CFPAC-1 cells treated with miR-194-5p antagomir or antagomir NC were used to inject into the armpits of nude mice subcutaneously to establish xenograft tumor model. Four weeks after injection, the tumors of each group were collected (Fig. 5A). The tumor weights and volumes of the miR-194-5p overexpression group were significantly higher than those in the negative control group; while the tumor weights and volumes of the miR-194-5p knockdown group were significantly lower than those of the negative control group (Fig. 5B, C). Furthermore, we performed the hematoxylin–eosin (H&E) and immunohistochemical (IHC) staining analyses. The results showed that the Ki-67 positive cells were significantly increased in the miR-194-5p overexpression group as well as the expression of N-cadherin; the expression of P21 and E-cadherin were significantly decreased compared to the agomir NC group, and this situation was reversed in the miR-194-5p knockdown group (Fig. 5D). These results demonstrated that overexpression of miR-194-5p promotes the tumor growth and that knockdown of miR-194-5p inhibits tumor progression in vivo.

### SOCS2 is a target gene of miR-194-5p that is downregulated in PDAC

**Fig. 6:**
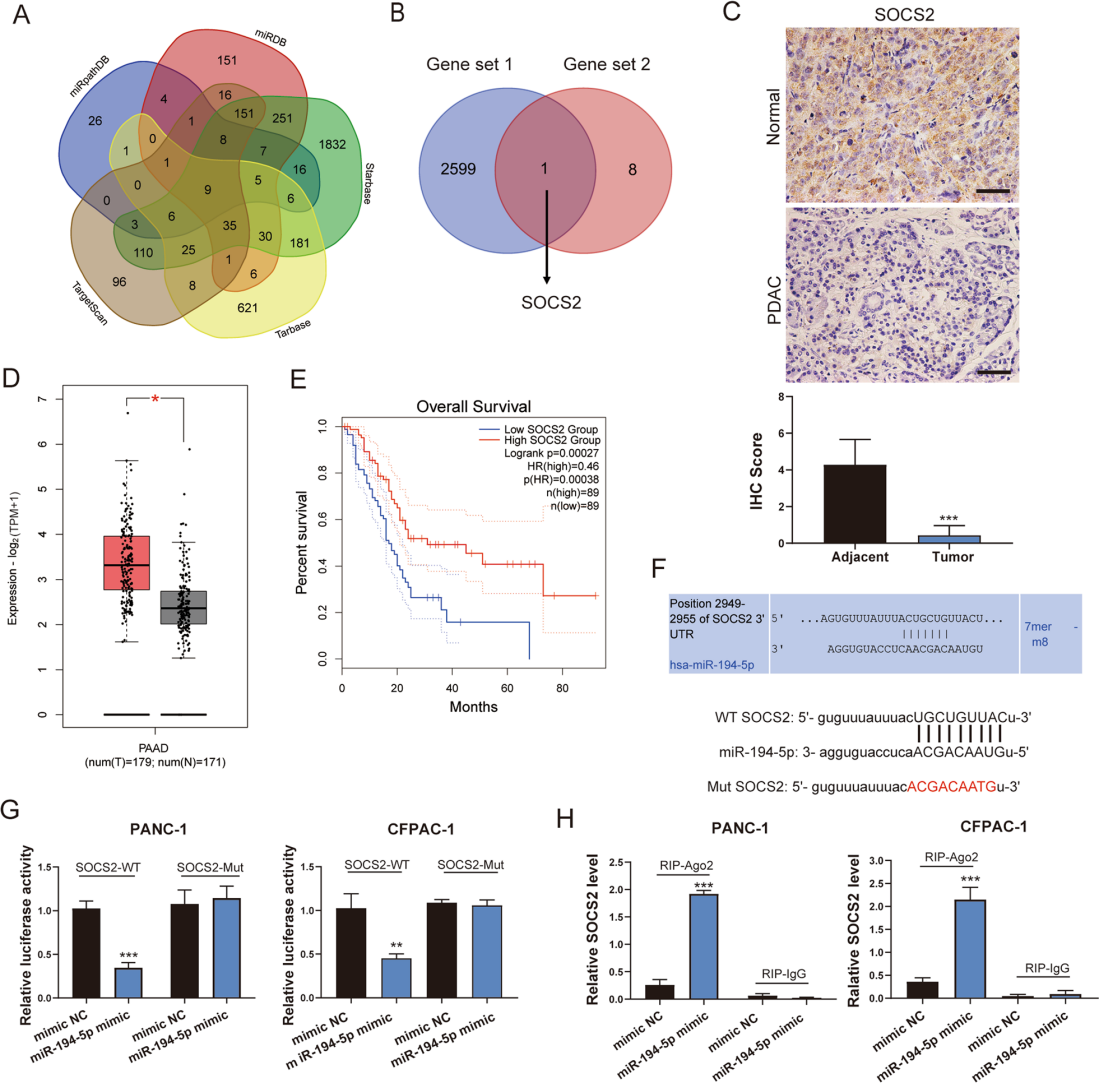
SOCS2 serves as a suppressor gene and a target of miR-194-5p. **A** The potential targets of miR-194-5p were predicted by miRDB, TargetScan, StarBase, Tarbase and miRpathDB. Nine target genes (Gene set 2) were obtained from the intersection. **B** The downregulated genes in PDAC of the GSE43795 dataset (Gene set 1) and the genes of Gene set 2 were intersected to obtain the target gene of miR-194-5p. **C** The expression of SOCS2 was obviously reduced in PDAC tissue compared with tumor-adjacent tissue, as shown by IHC staining (n = 6). Scale bar = 50 μm. **D** The expression of SOCS2 was decreased in PDAC compared tissue with normal tissue based on TCGA and GTEx data. **E** PDAC samples from the TCGA were divided into two groups (high and low SOCS2 expression groups). Kaplan‒Meier survival analysis was used to determine the overall survival rate. **F** The binding sites between miR-194-5p and SOCS2 mRNA were predicted by TargetScan (top). The WT and Mut sequences of the binding sites between SOCS2 mRNA and miR-194-5p were cloned into dual-luciferase reporter vectors (bottom). **G** A dual-luciferase reporter assay was performed by co-transfection of PDAC cells with WT/Mut SOCS2 plasmids and miR-194-5p mimic or mimic NC. **H** RIP and RT‒qPCR assays were performed to confirm the binding of miR-194-5p to the AGO2 protein. *p < 0.05; **p < 0.01; ***p < 0.001

To verify that miR-194-5p promotes tumor progression, we used both bioinformatic methods and mRNA-seq to identify SOCS2 as the target gene of miR-194-5p. First, we predicted miR-194-5p target genes by using several target gene prediction websites: TargetScan (http://www.targetscan.org/vert_72/), miRDB (http://mirdb.org/), Starbase (http://starbase.sysu.edu.cn), Tarbase (https://dianalab.e-ce.uth.gr/tools) and miRPathDB (https://mpd.bioinf.uni-sb.de/). The target genes from these databases were taken as the intersection and 9 target genes were obtained (Fig. 6A). Moreover, downregulated genes in PDAC from the GSE43795 dataset from the Gene Expression Omnibus (GEO) database were selected for further screening. Finally, one gene, SOCS2, was obtained. (Fig. 6B).

Herein, we detected the expression of SOCS2 in tumor-adjacent tissues and PDAC tissues via IHC. The results showed that SOCS2 was significantly downregulated in PDAC tissue (Fig. 6C). These results were consistent with the expression of SOCS2 from TCGA and GTEx data analysis with GEPIA (http://gepia.cancer-pku.cn/) (Fig. 6D). In addition, the expression of SOCS2 in PDAC cell lines was examined by RT‒qPCR, interestingly, the cell lines with low expression of SOCS2 were highly expressed miR-194-5p (Additional file 3: Fig S2A). Furthermore, we found that SOCS2 was significantly downregulated in the miR-194-5p overexpression group according to the mRNA-seq data (Additional file 3: Fig. S2D). The co-expression analysis indicated that SOCS2 had a negative relationship with miR-194-5p based on TCGA data by using StarBase (Additional file 3: Fig S2B). Kaplan–Meier survival analysis revealed that patients with low levels of SOCS2 had poor overall survival times according to TCGA data (Fig. 6E). The expression of SOCS2 in clinical stages showed a sharp reduction in stage II compared with other stages following the PDAC progression (Additional file 3: Fig. S2C).

Based on these findings, we predicted that SOCS2 was the target gene of miR-194-5p. To further verify this hypothesis, we investigated the binding sites between SOCS2 mRNA and miR-194-5p on the TargetScan website and constructed the WT and Mut luciferase reporter plasmids to detect the role of miR-194-5p in the regulation of SOCS2 mRNA activity (Fig. 6F). The results of the dual-luciferase reporter assay revealed that overexpression of miR-194-5p significantly reduced the luciferase activity of WT SOCS2 mRNA but not Mut SOCS2 mRNA after transfection (Fig. 6G). The RIP assay also confirmed that SOCS2 mRNA was preferentially enriched in miRNA-Ago2 ribonucleoprotein complexes (Fig. 6H). In addition, the expression of SOCS2 was reduced in the miR-194-5p agomir group, which was opposite to the miR-194-5p antagomir group by IHC staining (Additional file 4: Fig. S3C). Taken together, these results showed that miR-194-5p acts as a sponge for SOCS2 which acts as a tumor suppressor gene downregulated in PDAC.

### SOCS2 inhibits the proliferation and migration of PDAC cells via the PI3K/AKT signaling pathway

**Fig. 7:**
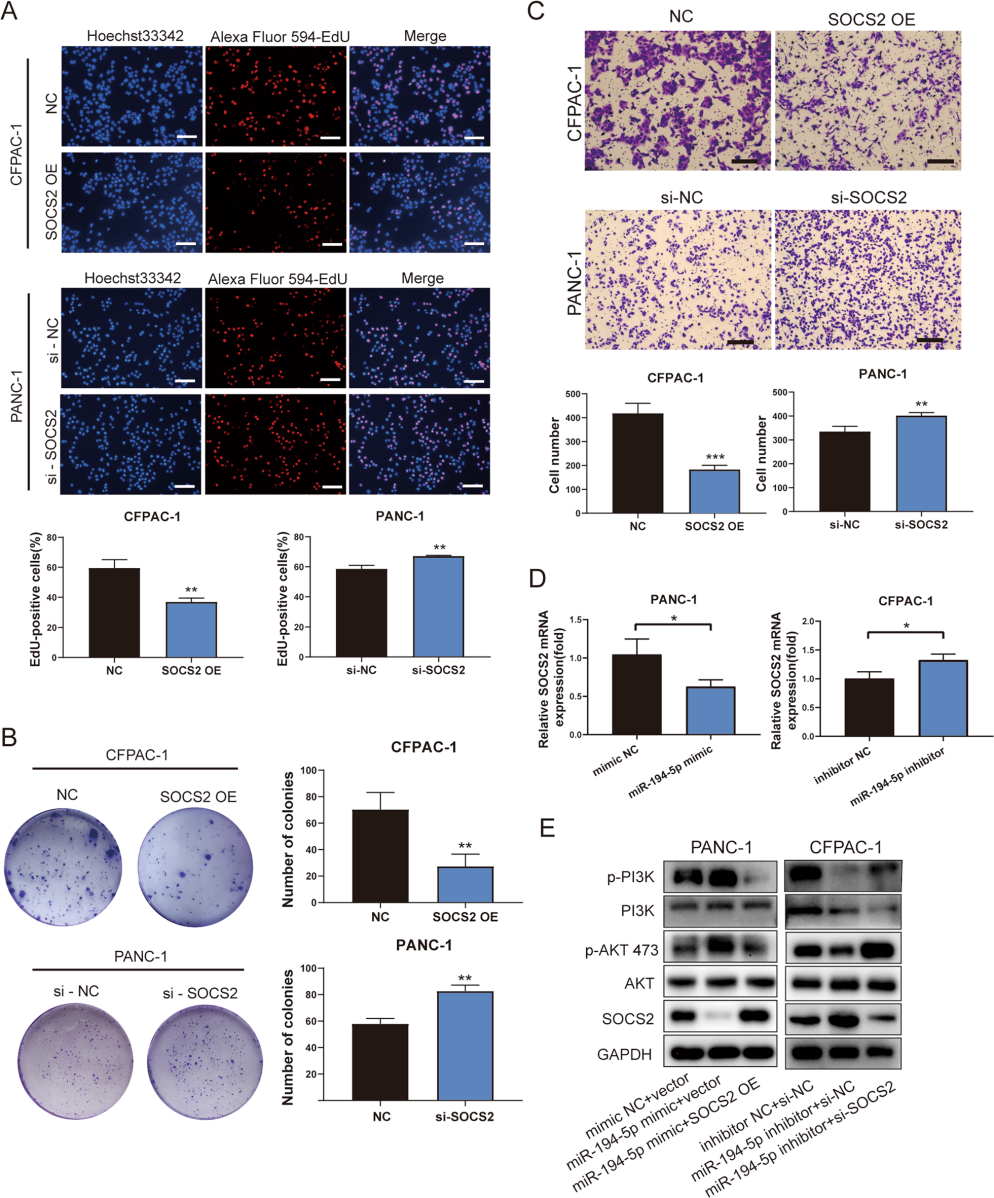
SOCS2 inhibits the PDAC cell proliferation and negatively regulates the activity of PI3K/AKT signaling pathway. **A**, **B** EdU and colony formation assays were performed to detect the proliferation ability of SOCS2 in PDAC cells. Scale bar = 100 μm. **C** Transwell assays were performed to observe the migration of PDAC cells after treatment with SOCS2 overexpression plasmids or si-SOCS2. Scale bar = 200 μm. **D** The level of SOCS2 in the miR-194-5p overexpression or knockdown group was determined by RT‒qPCR. **E** The activation level of the PI3K/AKT signaling pathway was observed in the groups with miR-194-5p mimic and SOCS2 overexpression alone or combined and the groups with miR-194-5p inhibitor and si-SOCS2 alone or combined by western blotting. *p < 0.05; **p < 0.01; ***p < 0.001

To clarify the function of SOCS2 in PDAC, we constructed the SOCS2 overexpression plasmid and small interfering RNA of SOCS2 to overexpress or knockdown the expression of SOCS2. The transfection efficiency was evaluated by RT‒qPCR and western blotting (Additional file 3: Fig. S2E, F). Next, we detected the biological function of SOCS2 in PDAC cells Subsequently, we performed the EdU, colony formation, and Transwell assays. These results indicated that overexpression of SOCS2 inhibited cell proliferation and migration (Fig. 7A–C). The changes of SOCS2 expression were opposite to the expression of miR-194-5p (Fig. 7D). Rescue experiments confirmed that SOCS2 could reverse the effect of miR-194-5p in cell proliferation and migration (Additional file 4: Fig. S3A, B). Previous studies revealed that the PI3K/AKT signaling pathway played an important role in tumors. SOCS2 was an inhibitor of the JAK/STAT signaling pathway, which is upstream of the PI3K/AKT signaling pathway. Therefore, the function of miR-194-5p was realized probably by absorbing SOCS2 mRNA to a large extent. To validate this hypothesis, we performed rescue experiments in CFPAC-1 and PANC-1 cells to assess the expression and phosphorylation levels of key factors of the PI3K/AKT signaling pathway. The results showed that overexpression of miR-194-5p increased the levels of PI3K and AKT phosphorylation in PANC-1 cells, and this effect was reversed by overexpression of SOCS2. However, the expression of p-PI3K and p-AKT showed a contrary tendency in CFPAC-1 cells treated with the miR-194-5p inhibitor (Fig. 7E). Taken together, these results demonstrate that SOCS2 is absorbed by miR-194-5p and simultaneously activates the PI3K/AKT signaling pathway.

### CircPVRL3 targets miR-194-5p directly and negatively correlates with the expression of miR-194-5p

**Fig. 8:**
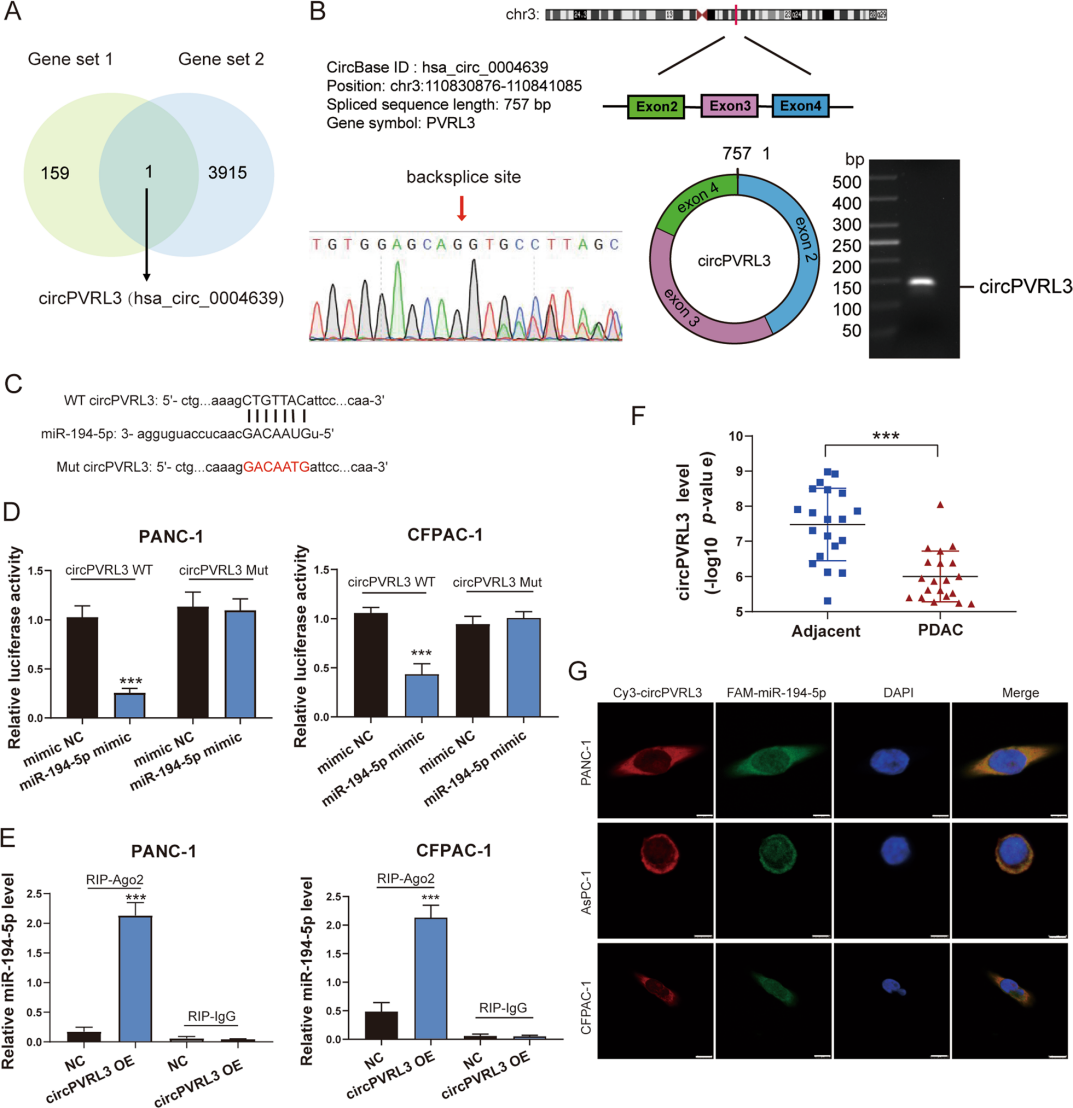
CircPVRL3, as a miR-194-5p sponge, is downregulated in PDAC. **A** The downregulated circRNAs of GSE79634 in PDAC tissue (Gene set 1) and the potential target of miR-194-5p were predicted by CircBase (Gene set 2) and intersected to obtain the upstream gene of miR-194-5p. **B** The circPVRL3 information is illustrated from CircBase, and the backsplice site indicates Sanger sequencing. **C** WT and Mut circPVRL3 were constructed and showed the binding sites between miR-194-5p and circPVRL3. **D** A dual-luciferase reporter assay was performed in PDAC cells co-transfected with WT/Mut circPVRL3 plasmids and miR-194-5p mimic or mimic NC. **E** RIP and RT‒qPCR assays were performed to confirm the binding of miR-194-5p to the AGO2 protein. **F** The expression of circPVRL3 in 20 paired PDAC tissues and adjacent tissues was detected by circRNA profiling. **G** The co-localization of Cy3-labeled circPVRL3 and FAM-labeled miR-194-5p was detected by confocal microscopy at a magnification of 1000×. Scale bar = 5 μm

To further explore the underlying molecular mechanism of miR-194-5p, we used circRNA sequencing data (GSE79634) from a circRNA microarray that contained 20 pairs of PDAC tissues and tumor-adjacent tissues, to investigate the upstream regulation of miR-194-5p. We found that there were 160 downregulated circRNAs in PDAC tissues, and the 3916 targeted circRNAs binding with miR-194-5p were predicted by CircBase [40]. The genes from two gene sets were intersected to obtain one circRNA, circPVRL3(hsa_circ_0004639) (Fig. 8A). Bioinformatics analysis showed that circPVRL3 was cyclized with the second to fourth exons of the PVRL3 gene. To examine the expression of circPVRL3, we first designed specific PCR primers for circPVRL3 to find the backsplice site, which was verified through Sanger sequencing (Fig. 8B). The presence of circPVRL3 was validated with divergent primers to identify backsplice junctions, and the presence of linear mRNAs was verified with convergent primers (Additional file 7: Fig. S5A). Subsequently, to verify the stability of circPVRL3, we performed an RNase R digestion experiment and analyzed the PCR products by agarose gel electrophoresis (Additional file 7: Fig. S5B). The results revealed that RNase R failed to digest circPVRL3, but dramatically decreased PVRL3 mRNA expression.

To verify the relationship between circPVRL3 and miR-194-5p, we constructed WT and Mut circPVRL3 (Fig. 8C). Then we performed a dual-luciferase reporter assay and RIP experiment. The results indicated that circPVRL3 regulated the expression of miR-194-5p by absorbing it directly (Fig. 8D, E). In addition, we performed a FISH experiment to observe the co-localization of circPVRL3 and miR-194-5p in PDAC cells. The results showed that circPVRL3 was mainly expressed in the cytoplasm; miR-194-5p was mostly expressed in the cytoplasm, and some were expressed in the nucleus, which provided a guarantee for operating the mechanism of ceRNA (Fig. 8G).

### CircPVRL3 is downregulated in PDAC and impacts proliferation and migration by competing with miR-194-5p in vitro and in vivo

**Fig. 9:**
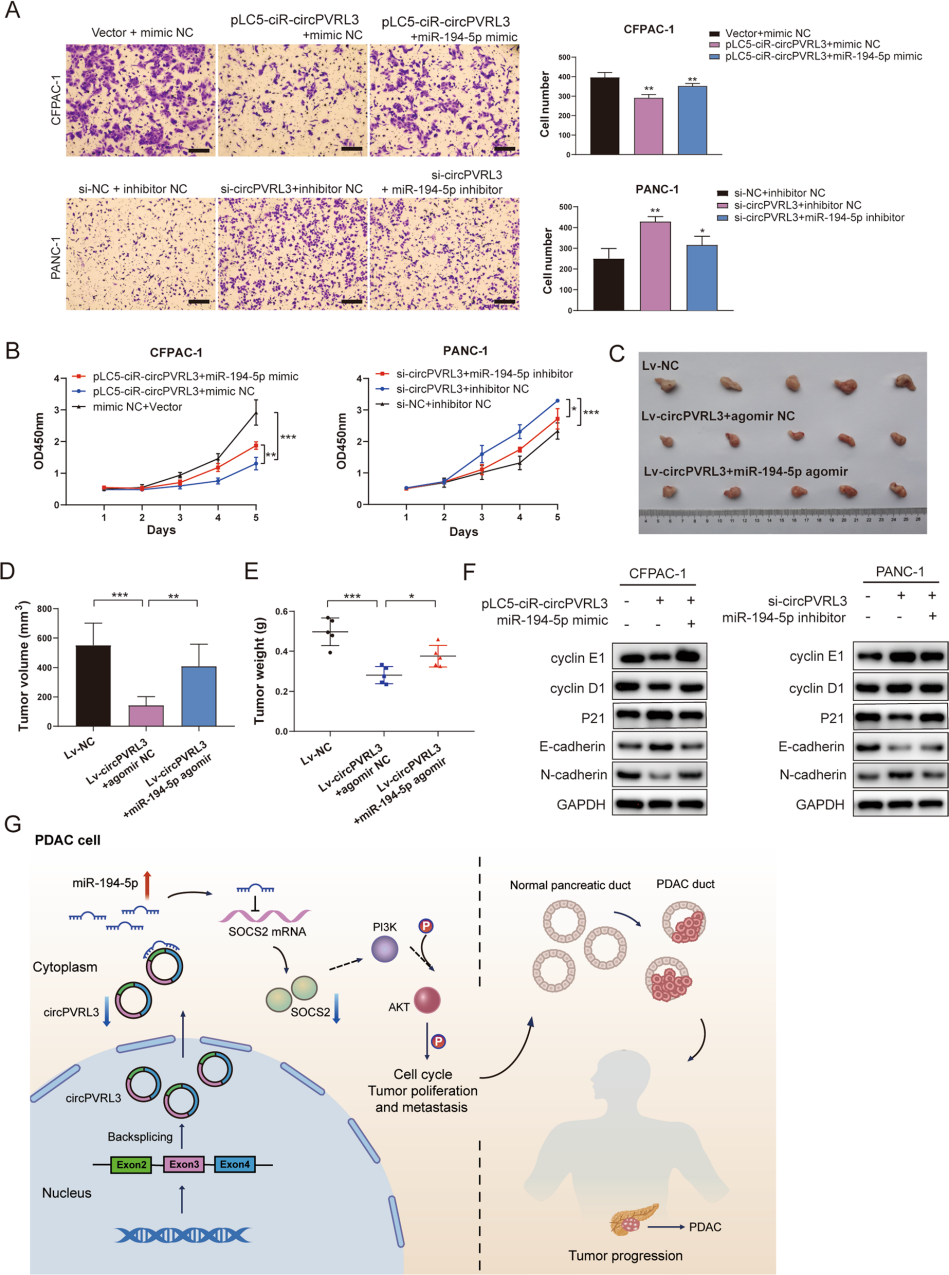
CircPVRL3 inhibits proliferation and migration through the miR-194-5p/SOCS2 axis in PDAC. **A** Transwell assays were performed to detect the effect of circPVRL3 on PDAC cell migration. Scale bar = 200 μm. **B** CCK-8 assay indicated the proliferation effect of circPVRL3 in PDAC cells. **C** CFPAC-1 cells were transduced with lentivirus to establish circPVRL3-overexpressing stable cells and injected subcutaneously in vivo. The effect of circPVRL3 was reversed by treatment with the miR-194-5p agomir. **D**, **E** The tumor volumes and weights were recorded (n = 5). **F** The key molecules of the PI3K/AKT signaling pathway and SOCS2 were detected by western blotting. **G** Schematic diagram showing how the circPVRL3/miR-194-5p/SOCS2 axis promotes PDAC by activating the PI3K/AKT signaling pathway. *p < 0.05; **p < 0.01; ***p < 0.001

According to the GEO dataset GSE79634, circPVRL3 was significantly downregulated in PDAC tissue (Fig. 8F). To clarify the biological function of circPVRL3, we performed proliferation assays. The results confirmed that overexpression of circPVRL3 suppressed the cell proliferation, and this effect was contrary to knockdown it (Additional file 5: Fig. S4A–C). CCK-8 and Transwell rescue experiments demonstrated that the effects of circPVRL3 could be reversed by miR-194-5p on the proliferation and migration of PDAC cells (Fig. 9A, B). These effects also reflect in the animal model, which showed that overexpression of circPVRL3 significantly reduced the tumor weight, volume, expression of Ki-67 and N-cadherin, and increased the expression of P21 and E-cadherin. These effects were also reversed by miR-194-5p (Fig. 9C–E, Additional file 7: Fig. S5D). Overexpression or knockdown of circPVRL3 could regulate the main biomarkers of proliferation and metastasis in CFPAC-1 and PANC-1 cells, and the miR-194-5p mimic or inhibitor rescued this regulation of circPVRL3 (Fig. 9F). Moreover, knockdown of circPVRL3 not only inhibited the expression of SOCS2 but also activated the PI3K/AKT signaling pathway, and overexpression of circPVRL3 induced the opposite effects (Additional file 7: Fig. S5C). In summary, these results indicated that low levels of circPVRL3 increased the expression of miR-194-5p by attenuating absorption of miR-194-5p which targets SOCS2 and activates the PI3K/AKT signaling pathway to promote the proliferation and migration of PDAC cells (Fig. 9G).

## Discussion

PDAC is a systemic disease prone to local progression and characteristic of metastasis. However, the pathogenesis mechanism of PDAC remains unclear. The commonly mutated genes in PDAC include KRAS, TP53, SMAD4, and CDKN2A, but related specific drugs are currently unavailable. The lack of specific targets and the toxic side effects of chemotherapy are obstacles to the treatment of PDAC [41, 42]. ncRNA is a core component of transcription, and miRNAs are posttranscriptional regulators that regulate nearly 50% of protein-coding genes [9]. In addition, miRNAs have shown promise and have provided an approach for the diagnosis and treatment of PDAC. miRNAs downregulate the expression of target genes by the sponge effect. Meanwhile, miRNAs are also subjected to the regulation of long non-coding RNAs (lncRNAs) or circRNAs simultaneously [25]. This ceRNA mechanism not only alters the temporal and spatial transcriptome but also plays an important role in tumor progression. In this study, we proposed that miR-194-5p expression at a high level, which is regulated by circPVRL3, promotes PDAC cell proliferation and migration by targeting SOCS2 and activating the PI3K/AKT signaling pathway.

Previous studies have reported that miR-194-5p is upregulated in PDAC and is related to PDAC tumor growth, which our findings are consistent with previous research. Studies revealed that miR-194-5p acts as a tumor promotor by targeting DACH1 [43]. Another study showed that lncRNA H19 could absorb miR-194-5p and upregulate PFTK1 through the WNT signaling pathway to promote the proliferation and migration of PDAC based on the lncRNA‒miRNA-mRNA regulatory network [44]. However, previous studies lacked verification with a large number of samples to some extent. In the present study, we used a tissue microarray containing 58 PDAC patient tissues, and six cell lines were used to examine the expression of miR-194-5p. We found that the expression of miR-194-5p was upregulated in PDAC tissue and two PDAC cell lines by RNAscope and RT‒qPCR. Next, we examined the function of miR-194-5p using gain-of-function and loss-of-function methods in vitro and in vivo. These results revealed that overexpressed miR-194-5p could accelerate the process of the cell cycle, and promote DNA replication and migration. Another study also reached a conclusion that was consistent with this study [45]. In the animal model, we found that overexpression of miR-194-5p also accelerated tumor growth and increased the percentage of Ki-67-positive cells verified by IHC. Therefore, miR-194-5p served as a tumor promotor in PDAC progression.

Subsequently, we further sought to identify the target gene of miR-194-5p and identified SOCS2 as the target gene that had been screened. Moreover, miR-194-5p combined with the 3’UTR of SOCS2 mRNA directly according to bioinformatics analysis and the results of mRNA-seq. The relationship between miR-194-5p and SOCS2 was confirmed by dual-luciferase reporter assay and RIP assay. Our results verified that overexpressed miR-194-5p sharply downregulated the level of SOCS2. Consequently, we intensively explored the regulatory mechanism of SOCS2. SOCS2 is one of the SOCS family members. Previous studies indicated that a decreased expression of SOCS2 was associated with the progression and poor prognosis of hepatocellular carcinoma (HCC) [46]. Similarly, SOCS2 was identified as a target of miR-196a and miR-196b that modulated the JAK/STAT signaling pathway affecting the progression of HCC [47]. Our results indicated that SOCS2 acted as a tumor suppressor gene in PDAC by inhibiting cell proliferation and migration by functional assays. Meanwhile, combined with SOCS2 is a negative regulator of the JAK/STAT signaling pathway which the PI3K/AKT signaling pathway is downstream of it. We predicted that SOCS2 affected biological function by regulating the PI3K/AKT signaling pathway. The PI3K/AKT signaling pathway is one of the essential pathways for regulating cell proliferation and the cell cycle and is critical in tumor progression [48, 49]. Our results showed that the phosphorylation levels of PI3K and AKT were enhanced by overexpressing miR-194-5p, which was opposite to the effect of knockdown miR-194-5p. Moreover, we observed that SOCS2 could partially reverse the expression of p-AKT and p-PI3K. Therefore, our results indicated that the activity of the PI3K/AKT signaling pathway was stimulated by miR-194-5p.

CircRNAs are one of the ncRNAs that are indispensable for tumorigenesis and tumor progression. Generally, circRNAs exert effects by absorbing target miRNAs, which is an important way to modulate tumor progression. The importance of circRNAs in the PI3K/AKT signaling pathway has been demonstrated in many studies. CircRNA-miRNA‒mRNA is a core regulatory network in different types of cancer. A previous study showed that circRNAs targeting different miRNAs regulated cancer progression via the PI3K/AKT signaling pathway mostly through this regulatory axis [50]. In colorectal cancer, circIL4R targeting miR-761 promoted proliferation and metastasis by activating the PI3K/AKT signaling pathway [51]. In osteosarcoma, circ_001422 targeted miR-195-5p and promoted the progression and metastasis through the miR-195-5p/FGF2/PI3K/Akt axis [52]. To explore the underlying molecular mechanism of miR-194-5p, we used previous circRNA profiling data and bioinformatics methods to identify the target of miR-194-5p. Finally, we obtained circPVRL3 (hsa_circ_0004639), which was derived from the exons 2,3 and 4 of the parental gene PVRL3. Our results demonstrated that circPVRL3 could inhibit tumor proliferation and migration in vitro and in vivo. Similarly, the downregulation of circRNA_0066779, which is derived from the exons 4, 5, 6, and 7 of the PVRL3 gene, promoted the progression of gastric cancer cells by targeting several miRNAs [53]. The dual-luciferase and RIP assays showed that circPVRL3 could directly combine with miR-194-5p. The co-localization of circPVRL3 and miR-194-5p by FISH validated the existence of the circRNA-miRNA‒mRNA network. Rescue experiments revealed that circPVRL3 absorbs miR-194-5p directly and regulates SOCS2 to manipulate the phosphorylation level of the PI3K/AKT signaling pathway.

The subsequent metastasis function of miR-194-5p through the circPVRL3/miR-194-5p/SOCS2 axis requires to perform further validation in vivo in the future. Because miRNAs could be used as biomarkers [54], another deficiency is the significance of the clinical diagnosis of miR-194-5p among PDAC patients need to be verified. Meanwhile, one study indicated that exosomes derived from dying tumor cells in PDAC after radiotherapy delivered miR-194-5p and further regulated E2F3 to enhance the survival of residual tumor repopulating cells (TRCs) [55]. Another study predicted circulating MIR1307 as a potential biomarker of patients who may benefit from FOLFIRINOX [56]. Therefore, miR-194-5p may be a potential diagnostic or prognostic biomarker in serum or other body fluid samples.

## Conclusion

This study indicated that miR-194-5p expression is increased in PDAC due to the low level of circPVRL3, which promotes the proliferation and migration of PDAC cells through the miR-194-5p/SOCS2 axis and activates the PI3K/AKT signaling pathway to promote the progression of PDAC. Therefore, targeting miR-194-5p in PDAC may be a potential therapeutic target for PDAC in the future.

## Supplementary Information

**Additional file 1.** Additional tables.

**Additional file 2: Figure S1.** The expression validation of miR-194-5p and KEGG pathway analysis. (A) The expression of miR-194-5p-related genes was observed by RT‒qPCR in the NC group and groups treated with 50 nM miR-194-5p mimic or 200 nM miR-194-5p inhibitor in PANC-1 cells. (B) The image shows the clustering heatmap of DEGs by RNA sequencing in PANC-1 cells with treated miR-194-5p mimic or not. (C) The KEGG analysis showed that genes related to overexpression of miR-194-5p are related to the cell cycle and miRNAs in cancer based on the results of mRNA-seq.

**Additional file 3: Figure S2.** SOCS2 is negatively related to the miR-194-5p and its expression is decreased in PDAC progression. (A) The expression of SOCS2 in HPDE-C7 cells and PDAC cell lines was observed by RT‒qPCR. (B) SOCS2 expression was negatively related to miR-194-5p according to TCGA data by StarBase. (C) Violin plots indicating the expression of SOCS2 based on patient pathological stage by GEPIA. (D) The SOCS2 expression data were first log2(TPM + 1) transformed for differential analysis by mRNA-seq between the mimic NC and miR-194-5p mimic groups. (E) and (F) The transfection efficiency of SOCS2 expression alteration with overexpression plasmid and small interfering RNA of SOCS2 was validated by RT‒qPCR and western blotting.

**Additional file 4: Figure S3.** MiR-194-5p affects the expression of SOCS2 which reverses the effects of miR-194-5p on cell proliferation and migration. (A) and (B), the number of colonies or cells was counted in the control group, miR-194-5p mimic or miR-194-5p inhibitor group, miR-194-5p mimic or miR-194-5p inhibitor group and SOCS2 overexpression plasmid or si-SOCS2 co-transfected group by colony formation and Transwell assays. (C) HE staining (scale bar = 100 μm) and the expression of SOCS2 by IHC staining (scale bar = 50 μm) of tumors derived from the subcutaneous xenograft model. The results were evaluated by IHC scoring (n = 5).

**Additional file 5: Figure S4.** CircPVRL3 inhibits the cell proliferation in PDAC cells. (A) EdU assay, (B) colony formation assay, and (C) CCK-8 assay were performed to detect the function of circPVRL3 which effected on the cell proliferation in PANC-1 and CFPAC-1 cells. Scale bar = 100 μm. *p < 0.05; **p < 0.01; ***p < 0.001.

**Additional file 6. Same as Figure S4.** Uncropped original western blots.

**Additional file 7: Figure S5.** Identification of circPVRL3, and effects of circPVRL3 on proliferation, EMT and the activity of PI3K/AKT signaling pathway in PDAC. (A) The convergent and divergent primers were used for detecting the circular or linear form to verify that circPVRL3 was only amplified in cDNA used by RT‒qPCR and agarose gel electrophoresis. GAPDH served as a control. (B) Total RNA derived from PANC-1 cells was extracted and treated with or without RNase R. The relative RNA levels were examined after RT‒qPCR by agarose gel electrophoresis. (C) The changes of the phosphorylation level of the PI3K/AKT signaling pathway by western blotting. (D) HE staining of tumors derived from the subcutaneous xenograft model. Scale bar = 100 μm. The expressions of Ki-67, P21, E-cadherin and N-cadherin were determined by IHC staining. Scale bar = 50 μm. The analyses were evaluated by IHC scoring (n = 5).

## Data Availability

For all data requests, please contact the corresponding author.

## Abbreviations

miRNAs: MicroRNA
ncRNA: Non-coding RNA
PDAC: Pancreatic ductal adenocarcinoma
RT-qPCR: Real-time quantitative PCR
circRNA: Circular RNA
RIP: RNA-binding protein immunoprecipitation
ceRNA: Competing endogenous RNAs
RISC: RNA-induced silencing complex
SOCS2: Suppressor of cytokine signaling 2;
SOCS: Suppressor of cytokine signaling
MREs: MiRNA recognition elements
FBS: Fetal bovine serum
ATCC: American Type Culture Collection
MEM: Minimum essential medium
DMEM: Dulbecco’s modified Eagle’s medium
siRNA: Small interfering RNA
WT: Wild-type
Mut: Mutant
FISH: Fluorescence in situ hybridization
mRNA-seq: MRNA-sequencing
SD: Standard deviation
TCGA: The Cancer Genome Atlas dataset
GO: Gene ontology
KEGG: Kyoto Encyclopedia of Genes and Genomes
GSEA: Gene set enrichment analysis
GEO: Gene expression omnibus
EMT: Epithelial-mesenchymal transition
H&E: Hematoxylin–eosin
IHC: Immunohistochemical
lncRNAs: Long non-coding RNAs
HCC: Hepatocellular carcinoma
TRCs: Tumor repopulating cells
