# Supplementary material for: Increased expression of miR-194-5p through the circPVRL3/miR-194-5p/SOCS2 axis promotes proliferation and metastasis in pancreatic ductal adenocarcinoma by activating the PI3K/AKT signaling pathway
Source: Cancer Cell Int. 2022 Dec 20;22:415. doi: 10.1186/s12935-022-02835-0 (PMC9764499; doi:10.1186/s12935-022-02835-0)
Supplement: Supplementary file 1 — Additional file 1. Additional tables. [file 12935_2022_2835_MOESM1_ESM.docx]

Table S1 Clinical baseline data of tissue microarray

| **Clinicopathological feature** | **Number** | **% of patients** |
| --- | --- | --- |
| **Age（y）** |  |  |
| ≥60 | 42 | 60.0 |
| <60 | 28 | 40.0 |
| **Gender** |  |  |
| Female | 25 | 35.7 |
| Male | 45 | 64.3 |
| **Clinical stage** |  |  |
| Ⅰ | 39 | 55.7 |
| Ⅱ | 18 | 25.7 |
| Ⅲ | 7 | 10.0 |
| Ⅳ | 4 | 5.7 |
| Missing data | 2 | 2.9 |
| **T classification** |  |  |
| T1 | 3 | 4.3 |
| T2 | 50 | 71.4 |
| T3 | 17 | 24.3 |
| **N classification** |  |  |
| N0 | 51 | 72.9 |
| N1 | 17 | 24.2 |
| Missing data | 2 | 2.9 |
| **Metastasis** |  |  |
| Yes | 4 | 5.7 |
| No | 64 | 91.4 |
| Missing data | 2 | 2.9 |
| **Pathology grade** |  |  |
| Ⅰ | 7 | 10.0 |
| Ⅱ | 44 | 62.9 |
| Ⅲ | 19 | 27.1 |
| **Nerve invasion** |  |  |
| Yes | 22 | 31.4 |
| No | 46 | 65.7 |
| Missing data | 2 | 2.9 |
| **Vital states (at follow-up)** |  |  |
| Alive | 12 | 17.1 |
| Dead | 55 | 78.6 |
| loss to follow-up | 3 | 4.3 |

Table S2 Sequences of Real-time quantitative PCR primer

| Gene | Primer sequences |
| --- | --- |
| GAPDH | Forward: GGCAAATTCCATGGCACCGT |
|  | Reverse: TGGACTCCACGACGTACTCA |
| PVRL3 | Forward: ATCTGTGTGGAGCAGGTTGG |
|  | Reverse: GTAGGCTGAAATGTAGATGACTT |
| PVRL3 | Forward: CAAGGAGAATATCAGGGAAG |
| (Convergent) | Reverse: AGTTGTAGAGGACTGGGCAT |
| circPVRL3 | Forward: TGATGGAAATTGGTTTGTAGGA |
| (Divergent) | Reverse: TCCCCATACTGCTGTGACAT |
| SOCS2 | Forward: AACCGCTCTACACGTCAGCA |
|  | Reverse: TGGTAAAGGCAGTCCCCAGA |

| Name | siRNA sequences |
| --- | --- |
| si-SOCS2 | GAACGGCACTGTTCACCTT |
| si-circPVRL3 | AGCAGGTGCCTTAGCTGGA |

Table S3 siRNA sequences
