# Supplementary figures and images for: Increased expression of miR-194-5p through the circPVRL3/miR-194-5p/SOCS2 axis promotes proliferation and metastasis in pancreatic ductal adenocarcinoma by activating the PI3K/AKT signaling pathway
Source: Cancer Cell Int. 2022 Dec 20;22:415. doi: 10.1186/s12935-022-02835-0 (PMC9764499; doi:10.1186/s12935-022-02835-0)

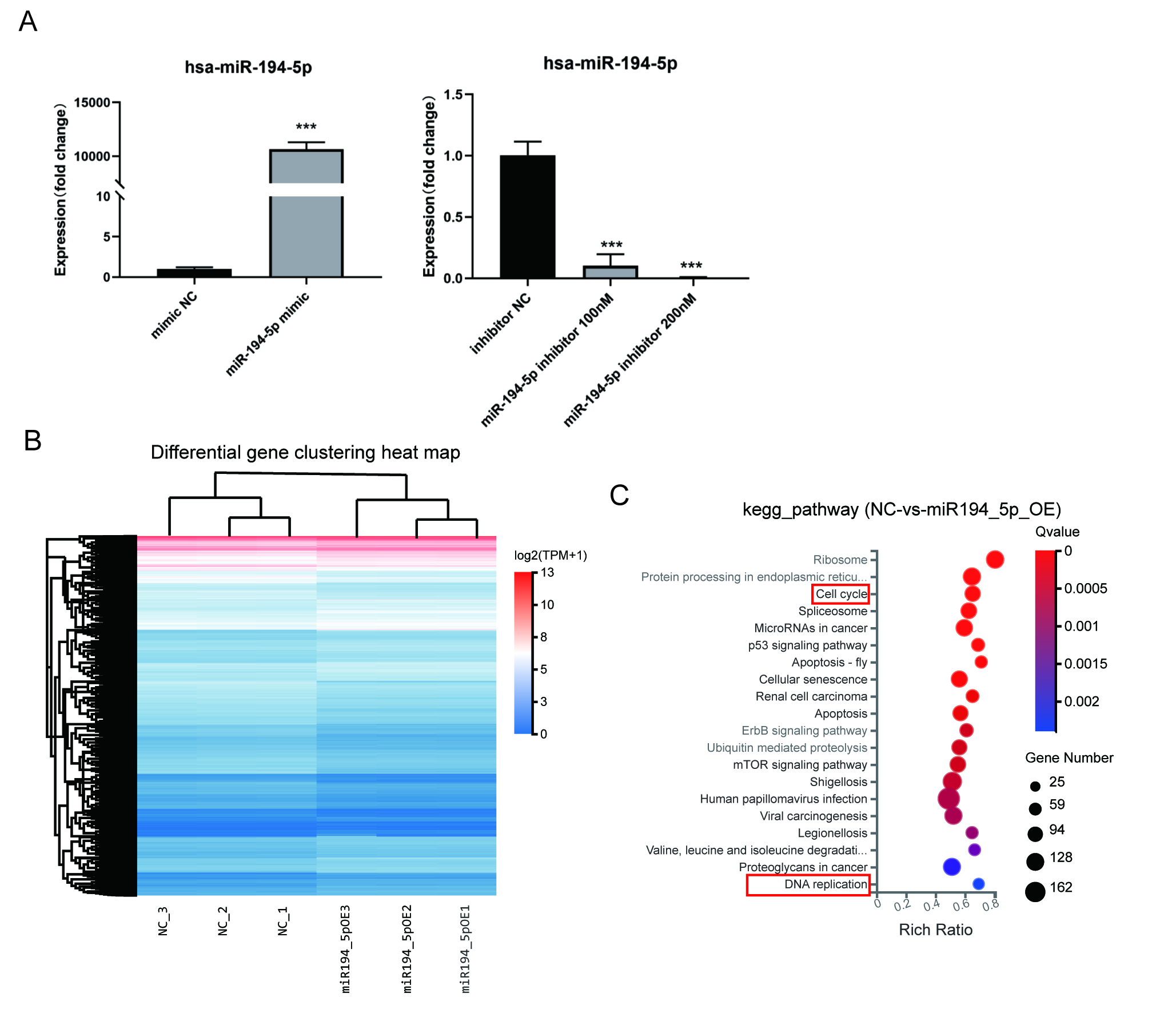

Supplement: Supplementary file 2 — Additional file 2: Figure S1. The expression validation of miR-194-5p and KEGG pathway analysis. (A) The expression of miR-194-5p-related genes was observed by RT‒qPCR in the NC group and groups treated with 50 nM miR-194-5p mimic or 200 nM miR-194-5p inhibitor in PANC-1 cells. (B) The image shows the clustering heatmap of DEGs by RNA sequencing in PANC-1 cells with treated miR-194-5p mimic or not. (C) The KEGG analysis showed that genes related to overexpression of miR-194-5p are related to the cell cycle and miRNAs in cancer based on the results of mRNA-seq. [file 12935_2022_2835_MOESM2_ESM.tif]

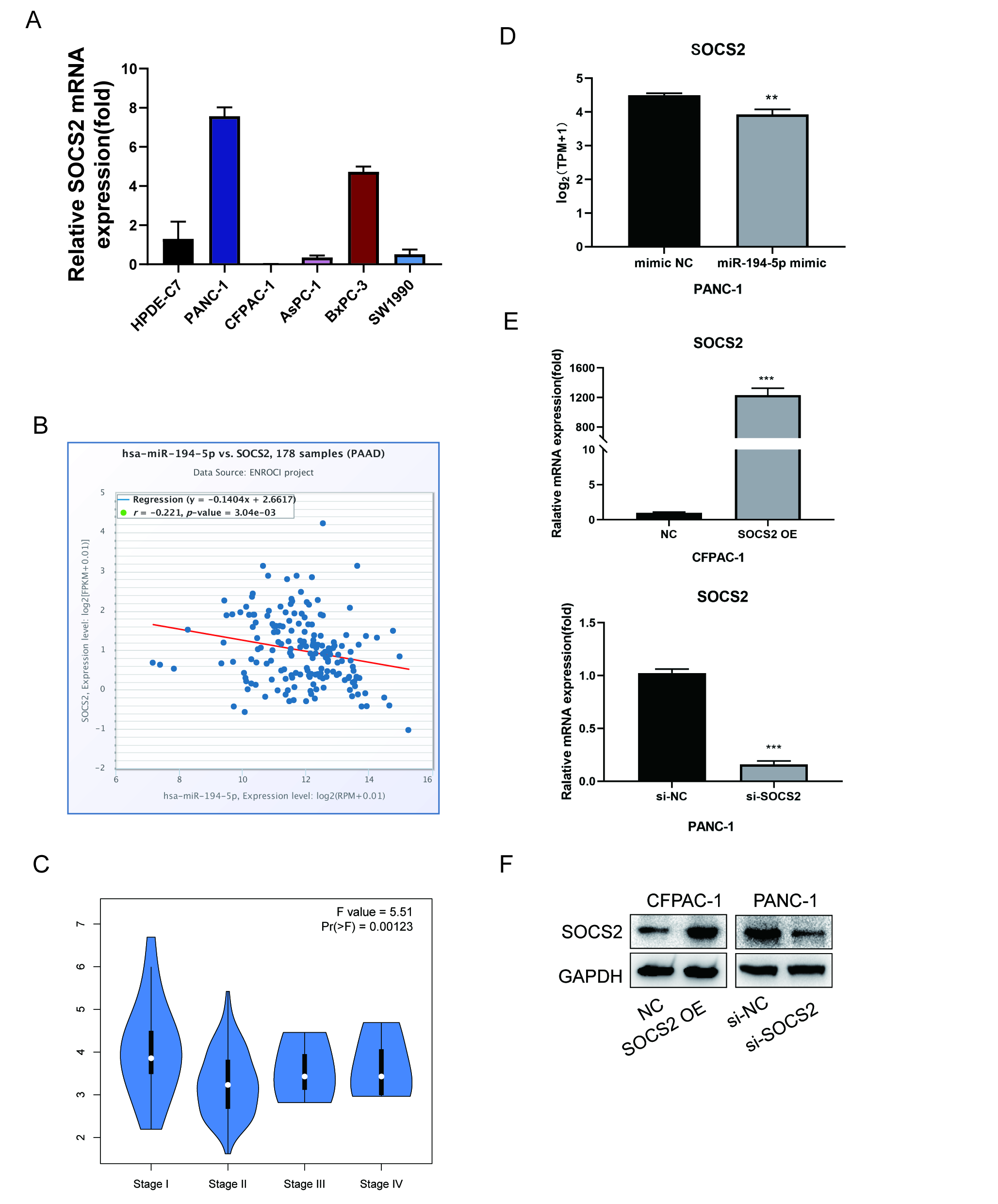

Supplement: Supplementary file 3 — Additional file 3: Figure S2. SOCS2 is negatively related to the miR-194-5p and its expression is decreased in PDAC progression. (A) The expression of SOCS2 in HPDE-C7 cells and PDAC cell lines was observed by RT‒qPCR. (B) SOCS2 expression was negatively related to miR-194-5p according to TCGA data by StarBase. (C) Violin plots indicating the expression of SOCS2 based on patient pathological stage by GEPIA. (D) The SOCS2 expression data were first log2(TPM + 1) transformed for differential analysis by mRNA-seq between the mimic NC and miR-194-5p mimic groups. (E) and (F) The transfection efficiency of SOCS2 expression alteration with overexpression plasmid and small interfering RNA of SOCS2 was validated by RT‒qPCR and western blotting. [file 12935_2022_2835_MOESM3_ESM.tif]

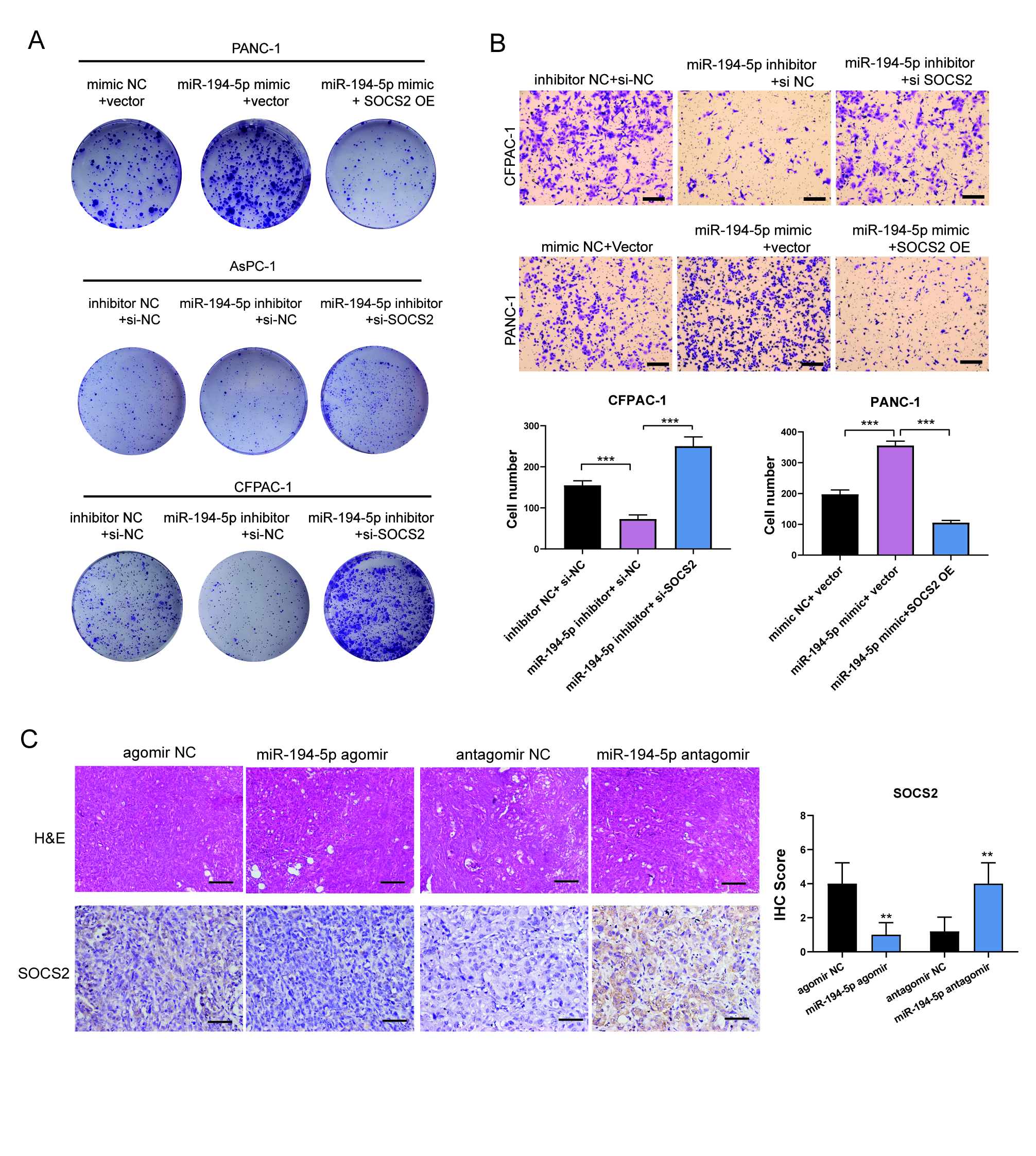

Supplement: Supplementary file 4 — Additional file 4: Figure S3. MiR-194-5p affects the expression of SOCS2 which reverses the effects of miR-194-5p on cell proliferation and migration. (A) and (B), the number of colonies or cells was counted in the control group, miR-194-5p mimic or miR-194-5p inhibitor group, miR-194-5p mimic or miR-194-5p inhibitor group and SOCS2 overexpression plasmid or si-SOCS2 co-transfected group by colony formation and Transwell assays. (C) HE staining (scale bar = 100 μm) and the expression of SOCS2 by IHC staining (scale bar = 50 μm) of tumors derived from the subcutaneous xenograft model. The results were evaluated by IHC scoring (n = 5). [file 12935_2022_2835_MOESM4_ESM.tif]

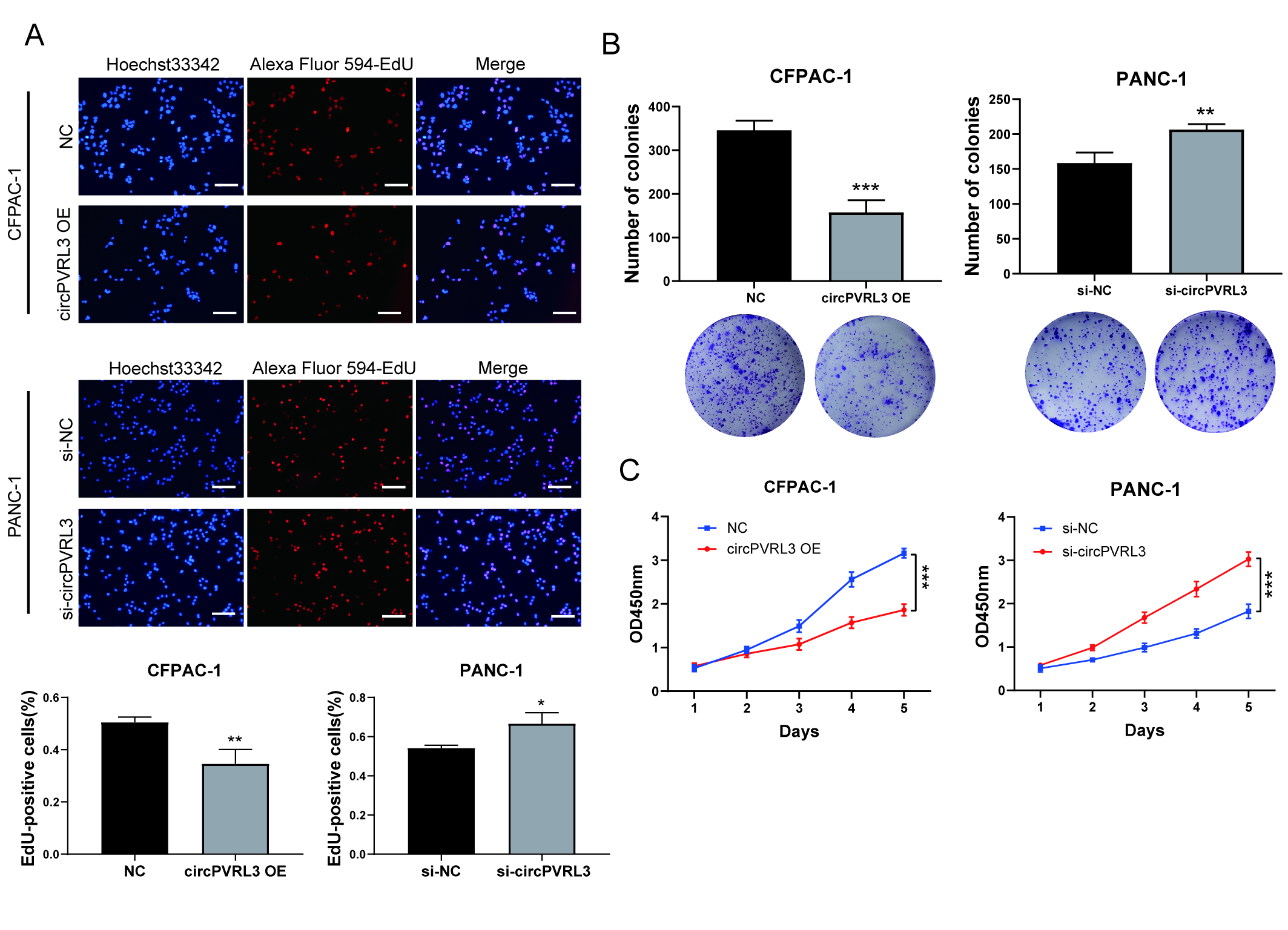

Supplement: Supplementary file 5 — Additional file 5: Figure S4. CircPVRL3 inhibits the cell proliferation in PDAC cells. (A) EdU assay, (B) colony formation assay, and (C) CCK-8 assay were performed to detect the function of circPVRL3 which effected on the cell proliferation in PANC-1 and CFPAC-1 cells. Scale bar = 100 μm. *p < 0.05; **p < 0.01; ***p < 0.001. [file 12935_2022_2835_MOESM5_ESM.tif]

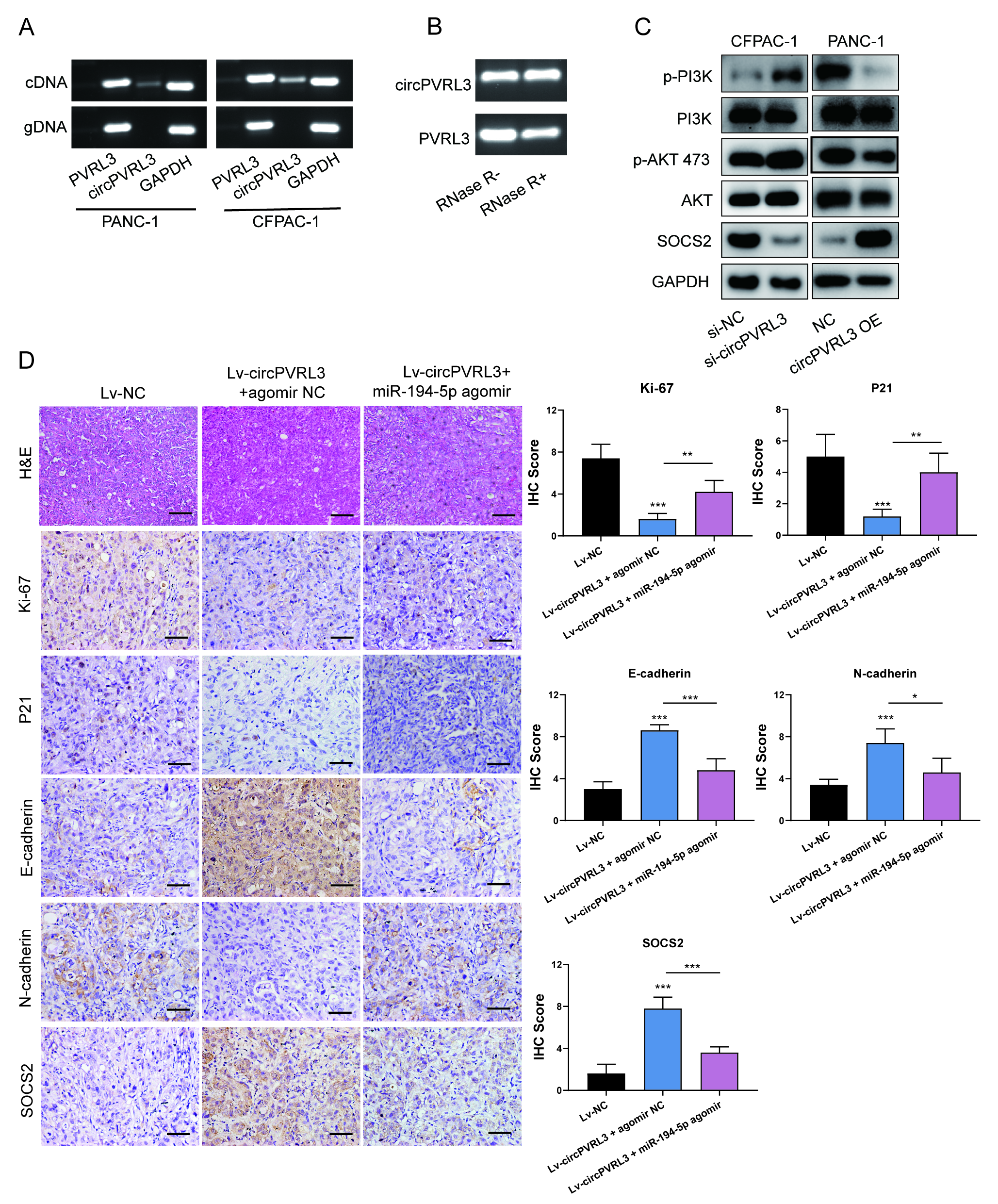

Supplement: Supplementary file 7 — Additional file 7: Figure S5. Identification of circPVRL3, and effects of circPVRL3 on proliferation, EMT and the activity of PI3K/AKT signaling pathway in PDAC. (A) The convergent and divergent primers were used for detecting the circular or linear form to verify that circPVRL3 was only amplified in cDNA used by RT‒qPCR and agarose gel electrophoresis. GAPDH served as a control. (B) Total RNA derived from PANC-1 cells was extracted and treated with or without RNase R. The relative RNA levels were examined after RT‒qPCR by agarose gel electrophoresis. (C) The changes of the phosphorylation level of the PI3K/AKT signaling pathway by western blotting. (D) HE staining of tumors derived from the subcutaneous xenograft model. Scale bar = 100 μm. The expressions of Ki-67, P21, E-cadherin and N-cadherin were determined by IHC staining. Scale bar = 50 μm. The analyses were evaluated by IHC scoring (n = 5). [file 12935_2022_2835_MOESM7_ESM.tif]
