# Supplementary material for: Increased expression of miR-194-5p through the circPVRL3/miR-194-5p/SOCS2 axis promotes proliferation and metastasis in pancreatic ductal adenocarcinoma by activating the PI3K/AKT signaling pathway
Source: Cancer Cell Int. 2022 Dec 20;22:415. doi: 10.1186/s12935-022-02835-0 (PMC9764499; doi:10.1186/s12935-022-02835-0)

Uncropped original western blots

Figure 4C

PANC-1 GAPDH

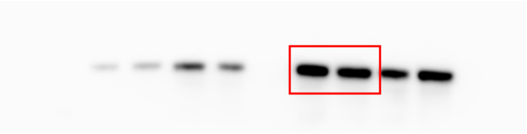

PANC-1 N-cadherin

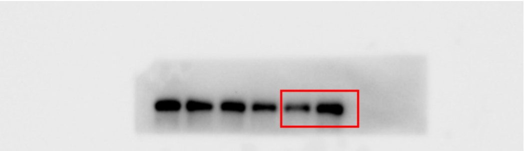

PANC-1 E-cadherin

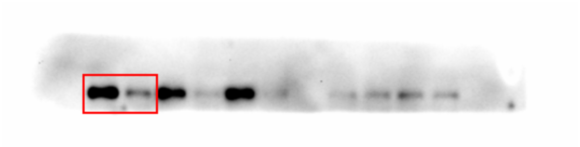

PANC-1 P21

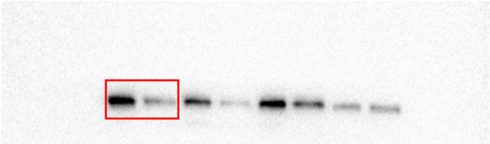

PANC-1 cyclin D1

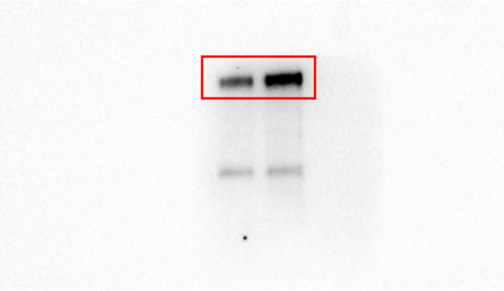

PANC-1 cyclin E1

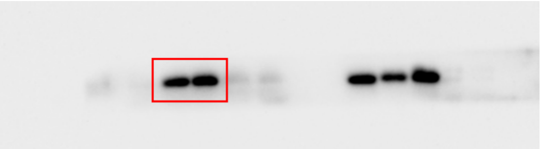

PANC-1 CDK2

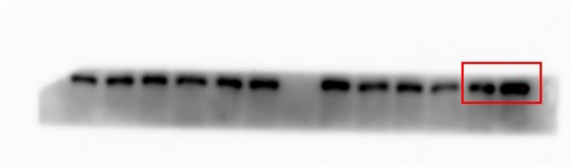

PANC-1 CDK4

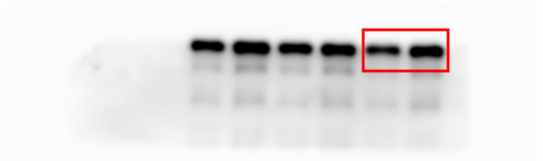

AsPC-1 GAPDH

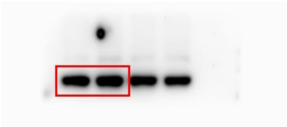

AsPC-1 N-cadherin

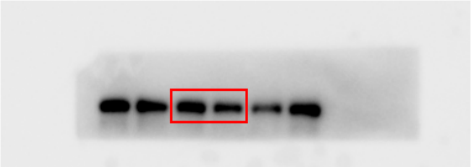

AsPC-1 E-cadherin

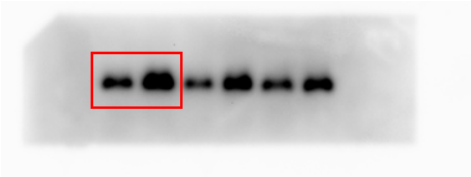

AsPC-1 P21

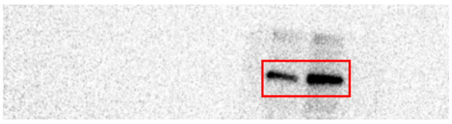

AsPC-1 cyclin D1

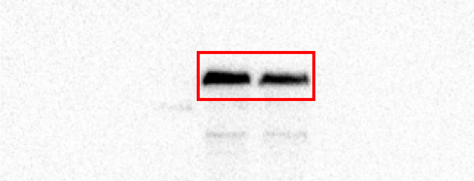

AsPC-1 cyclin E1

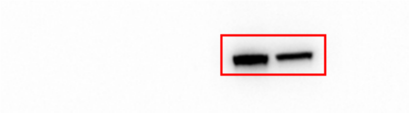

AsPC-1 CDK2

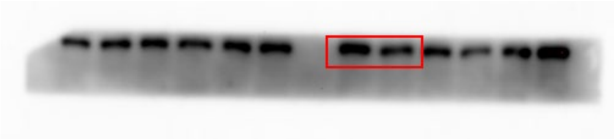

AsPC-1 CDK4

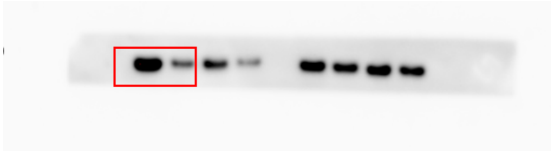

CFPAC-1 GAPDH

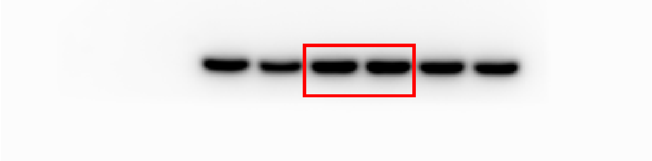

CFPAC-1 N-cadherin

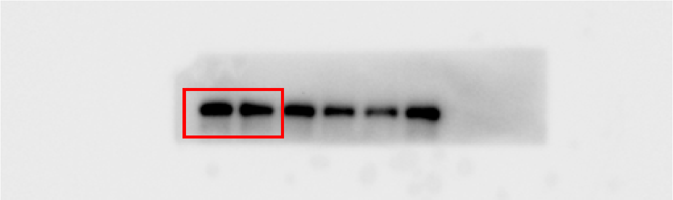

CFPAC-1 E-cadherin

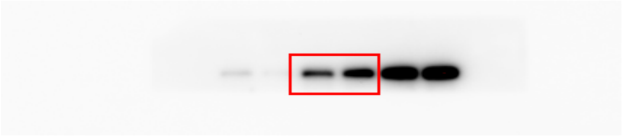

CFPAC-1 P21

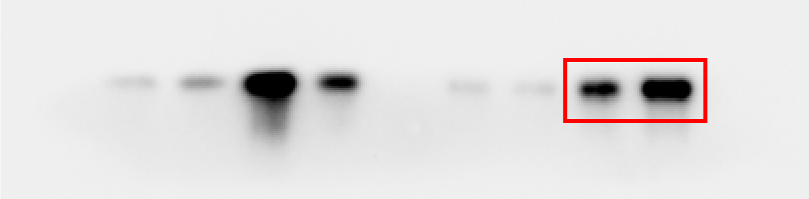

CFPAC-1 cyclin D1

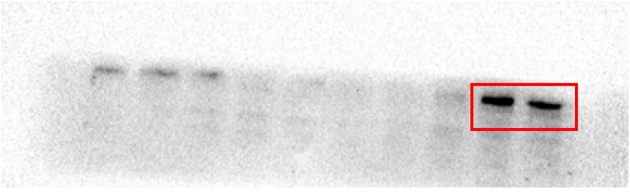

CFPAC-1 cyclin E1

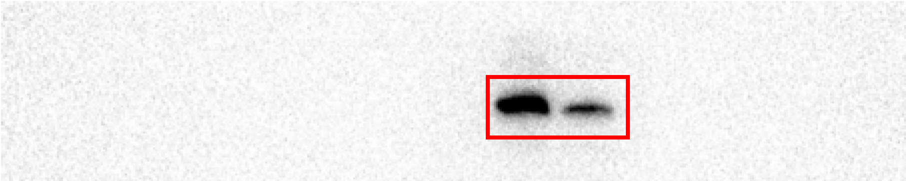

CFPAC-1 CDK2

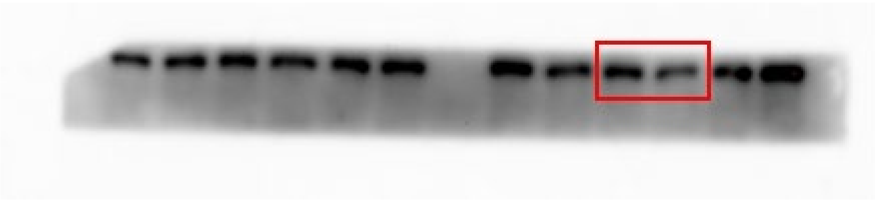

CFPAC-1 CDK4

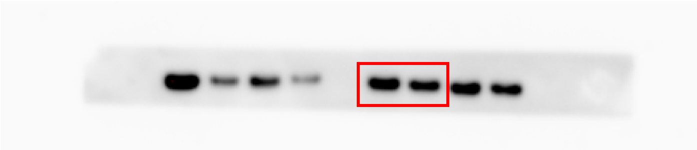

Figure 7E

PANC-1 GAPDH

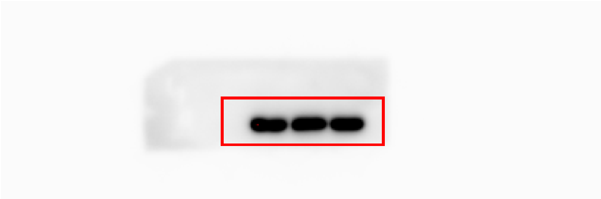

PANC-1 SOCS2

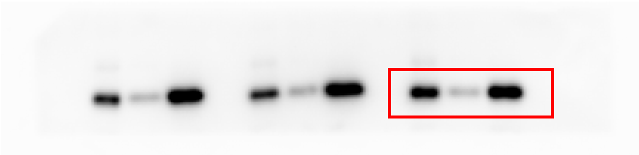

PANC-1 AKT

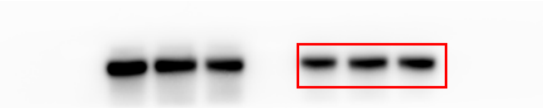

PANC-1 p-AKT 473

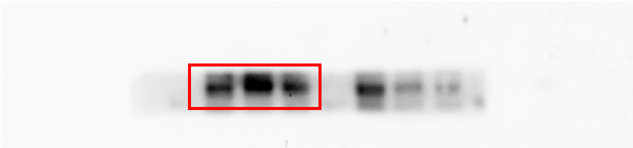

PANC-1 P13K

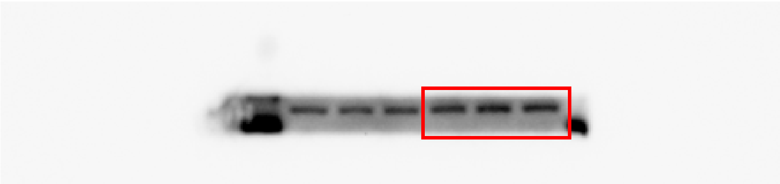

PANC-1 p-P13K

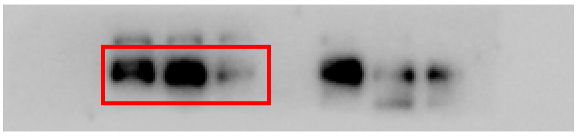

CFPAC-1 GAPDH

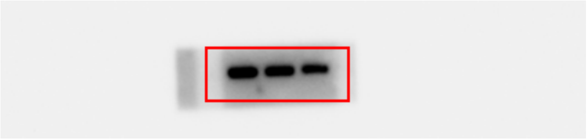

CFPAC-1 SOCS2

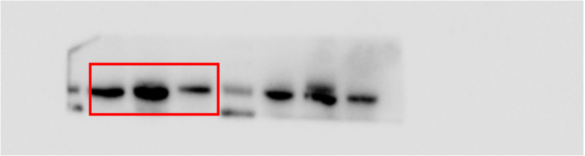

CFPAC-1 AKT

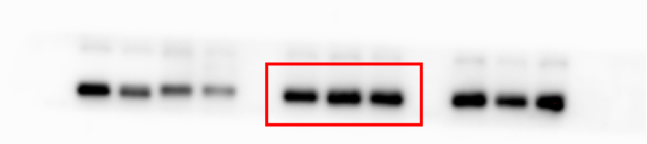

CFPAC-1 p-AKT 473

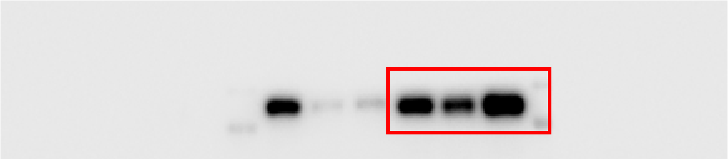

CFPAC-1 PI3K

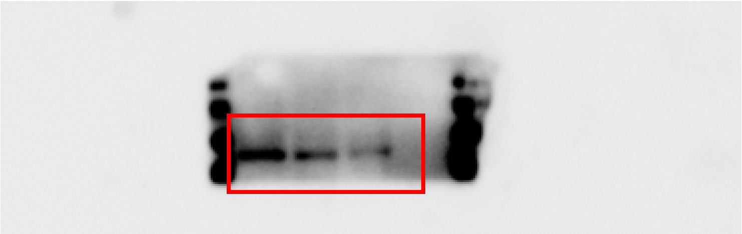

CFPAC-1 p-PI3K

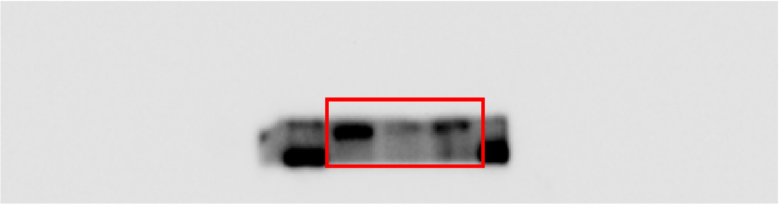

Figure 8B and Figure S3

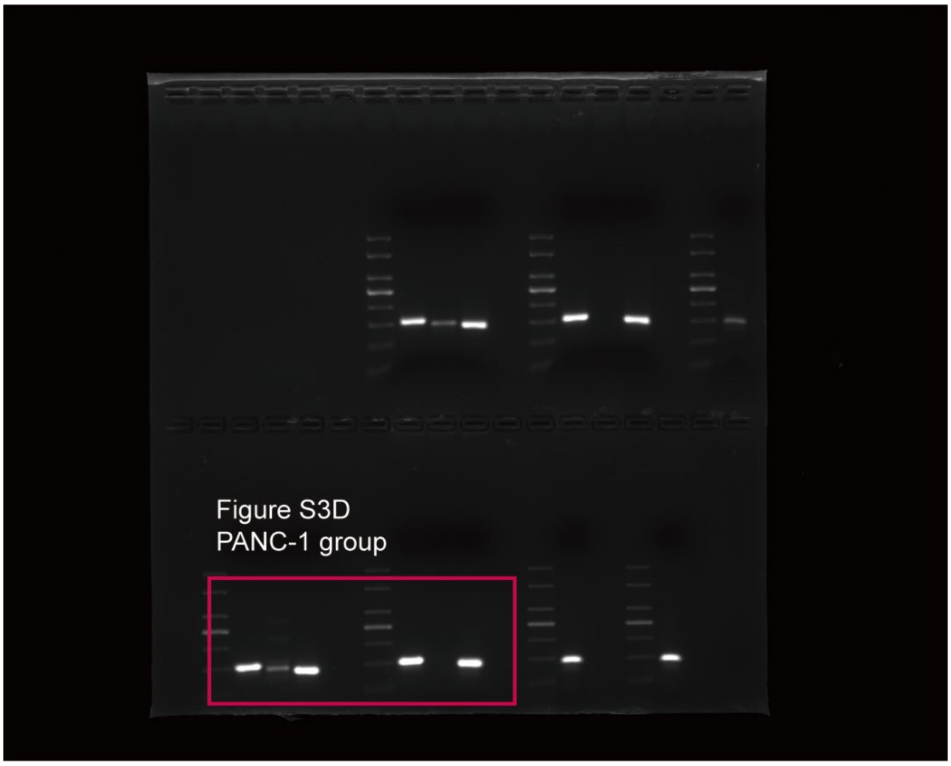

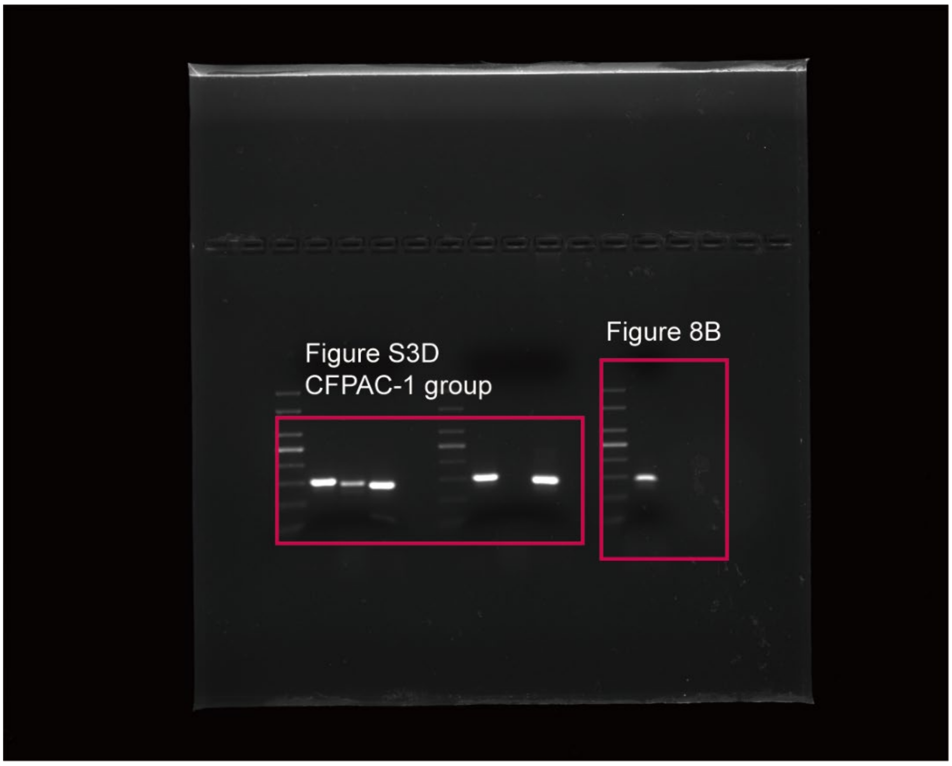

Figure 9F

CFPAC-1 GAPDH

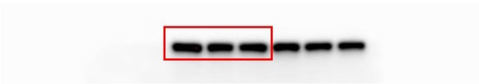

CFPAC-1 N-cadherin

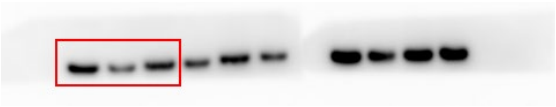

CFPAC-1 E-cadherin

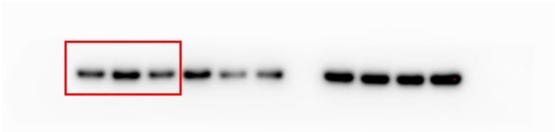

CFPAC-1 P21

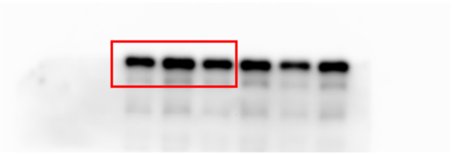

CFPAC-1 cyclinD1

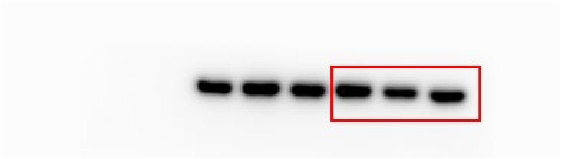

CFPAC-1 cyclinE1

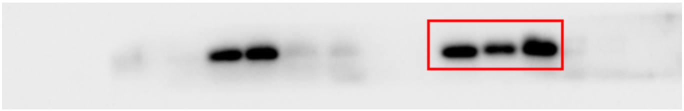

PANC-1 GAPDH

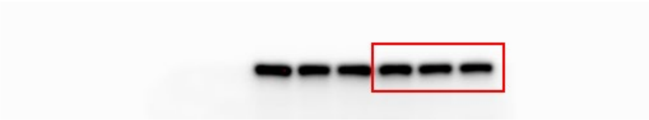

PANC-1 N-cadherin

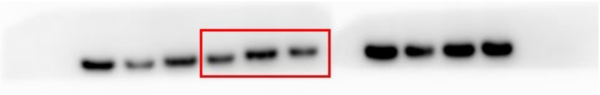

PANC-1 E-cadherin

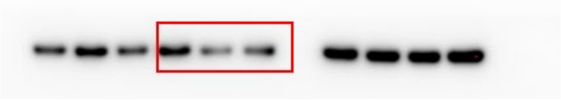

PANC-1 P21

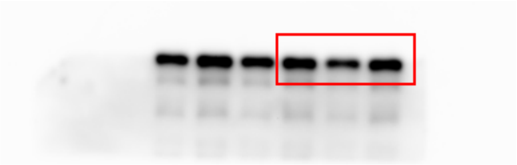

PANC-1 cyclinD1

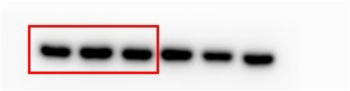

PANC-1 cyclinE1

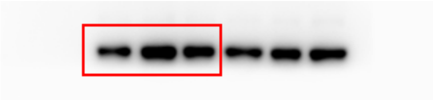

Figure S2F

CFPAC-1 GAPDH

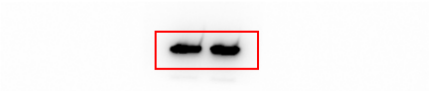

CFPAC-1 SOCS2

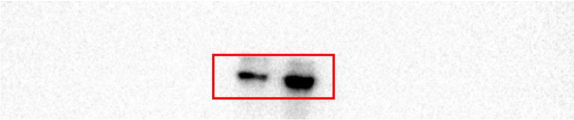

PANC-1 GAPDH

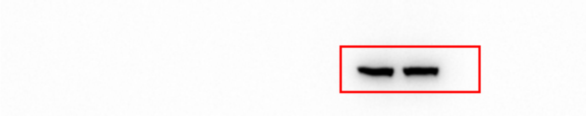

PANC-1 SOCS2

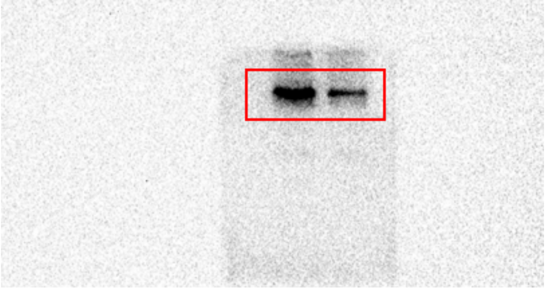

Figure S3F

CFPAC-1 GAPDH

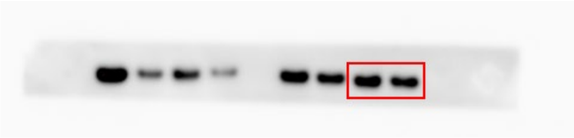

CFPAC-1 SOCS2

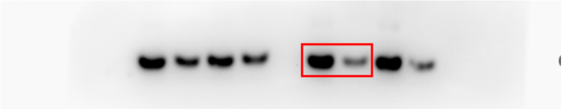

CFPAC-1 AKT

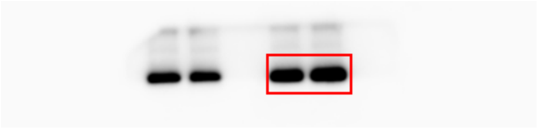

CFPAC-1 p-AKT

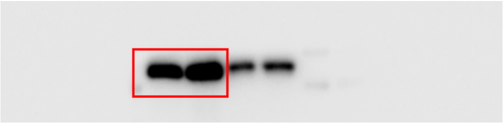

CFPAC-1 PI3K

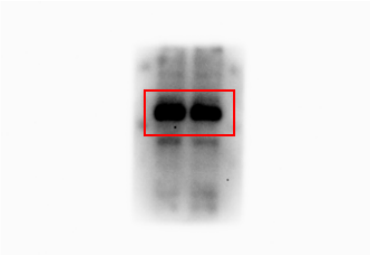

CFPAC-1 p-PI3K

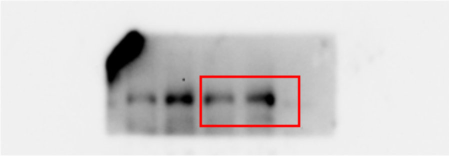

PANC-1 GAPDH

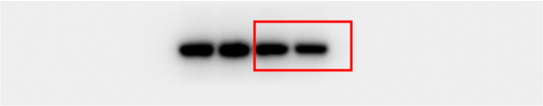

PANC-1 SOCS2

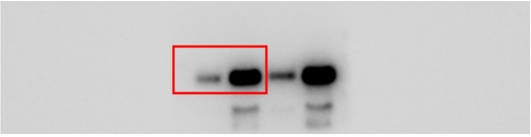

PANC-1 AKT

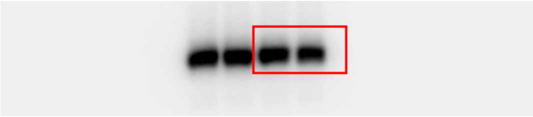

PANC-1 p-AKT

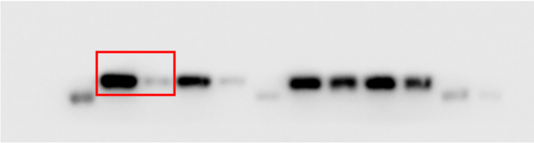

PANC-1 PI3K

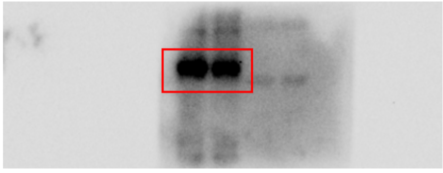

PANC-1 p-PI3K

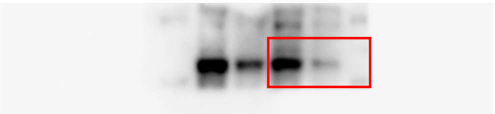

Supplement: Supplementary file 6 — Additional file 6. Same as Figure S4. Uncropped original western blots. [file 12935_2022_2835_MOESM6_ESM.pdf]
